# The endoplasmic reticulum as an active liquid network

**DOI:** 10.1073/pnas.2409755121

**Published:** 2024-10-11

**Authors:** Zubenelgenubi C. Scott, Samuel B. Steen, Greg Huber, Laura M. Westrate, Elena F. Koslover

**Affiliations:** ^a^Department of Physics, University of California, San Diego, La Jolla, CA 92093; ^b^Department of Chemistry and Biochemistry, Calvin University, Grand Rapids, MI 49546; ^c^Chan Zuckerberg Biohub—San Francisco, San Francisco, CA 94158

**Keywords:** endoplasmic reticulum, physical modeling, networks, organelle structure, subcellular dynamics

## Abstract

The peripheral endoplasmic reticulum (ER) forms a dense, interconnected, and constantly evolving network of membrane-bound tubules in eukaryotic cells. While individual structural elements and the morphogens that stabilize them have been described, a quantitative understanding of the dynamic large-scale network topology remains elusive. We develop a physical model of the ER as an active liquid network, governed by a balance of tension-driven shrinking and new tubule growth. This minimalist model gives rise to steady-state network structures with density and rearrangement timescales predicted from the junction mobility and tubule spawning rate. Several parameter-independent geometric features of the liquid network model are shown to be representative of ER architecture in live mammalian cells. The liquid network model connects the timescales of distinct dynamic features such as ring closure and new tubule growth in the ER. Furthermore, it demonstrates how the steady-state network morphology on a cellular scale arises from the balance of microscopic dynamic rearrangements.

## Significance

The peripheral endoplasmic reticulum (ER) forms a continuous, dynamic network of tubules that plays an important role in protein sorting, export and quality control, cellular signaling, and stress response. Elucidating how the unique morphology of the ER arises and supports its function is critical to developing a mechanistic understanding of the many neurological diseases associated with ER structural perturbations. We develop a physical model of the ER as an active liquid network, revealing how its cellular-scale structure emerges from small-scale dynamic rearrangements. The model demonstrates how key features of ER architecture can arise from a balance of tubule growth and tension-driven sliding. This work provides insight into the fundamental physical mechanisms underlying the emergent morphology of the ER.

The endoplasmic reticulum (ER) consists of a vast interconnected web of membrane-bound tubules and sheets in eukaryotic cells. It forms connections with many subcellular structures (1, 2), synthesizes and delivers lipids to other organelles (3, 4), stores and releases calcium (5, 6), and serves as a hub for the translation, folding, and quality control of secreted proteins (7, 8). The ER is highly dynamic and assumes a variety of structural motifs, which aid in accomplishing these diverse functional roles and maintaining its interconnection with other organelles (9).

Prior work on ER structure has focused on how its diverse morphologies [tubules, junctions, helicoidal ramps, fenestrated sheets, cisternae, etc. (10)] arise from an interplay of ER morphogen proteins and membrane mechanics. A variety of ER membrane proteins (including the reticulons and the DP1/REEP/Yop1p family) induce and stabilize the high positive curvature of tubules (11, 12). Others (e.g.: Climp63) stabilize the thickness of sheets and tubules (13, 14), while proteins such as lunapark (15) and the atlastin GTPase family (15–17) help to form and maintain junctions. It has been shown that the relative abundance of junctions, tubules, and sheets is determined by a combination of the absolute concentrations of curvature-producing proteins and membrane tension (13, 18). In other work, it was found that a diverse set of ER morphologies can be generated by tuning the proportions of reticulons and lunapark (19). Recently, a model was developed which highlighted the role of intrinsic membrane curvature and ultralow tensions in generating ER tubular matrices, ER sheet nanoholes, and other intricate membrane structures (20). These studies have helped elucidate the diverse structures observed in the ER as manifestations of local mechanical equilibrium.

Other work has sought to understand the link between ER dynamics and its structure. For instance, the anomalous diffusion of ER exit sites along tubules is captured by a model of an individual ER tubule as a semiflexible polymer (21). The fluctuations of individual tubules have also been quantified, with variations in their dynamic behavior associated with different regions of the cell (22). The dynamics of the ER are also implicated in controlling other subcellular structures; for instance, the motion of junctions may help regulate the distribution of microtubules within the cell (23).

Prior modeling work on plant cell ER (24, 25) demonstrated how small networks between persistent points appear to minimize tubule length, consistent with observations from in vitro studies of reconstituted lipid-protein systems (26). Although these studies focused on small regions of the ER with only a few tubules, quantification of such minimal networks enabled estimation of biophysical quantities such as local membrane tension and viscoelastic properties of the cytoplasm. Notably, the ER was treated not as a polymer chain with spring-like stretching or bending energies, but rather as a network with constant tension along effectively fluid tubules, an assumption which we also adopt in this work.

**Fig. 1.**
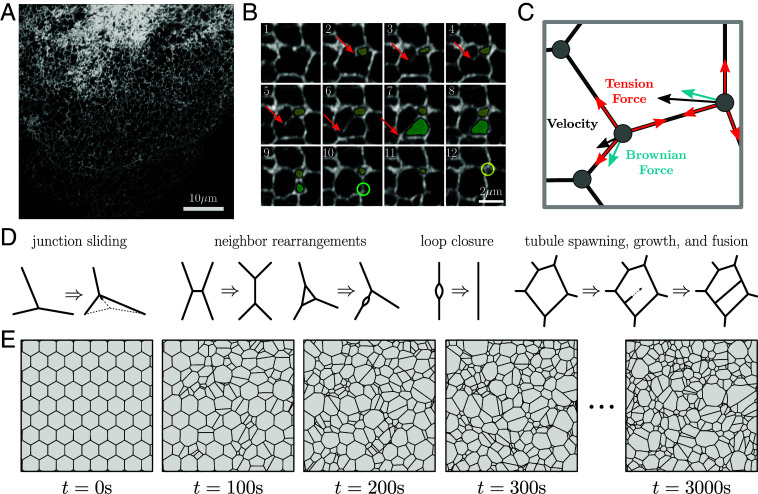
The dynamic structure of the endoplasmic reticulum is represented by a liquid network model. (*A*) Confocal image of COS7 cell expressing fluorescent endoplasmic reticulum (ER) marker (KDEL_mcherry), highlighting the peripheral ER network morphology. (*B*) Montage from the same cell line illustrating the spawning of a new tubule (red arrow) and loop closure events (yellow and green shaded regions). Size of each image is 5.0×5.0 μm with a time step of 0.6 s between each frame. (*C*) Physical model of junctions in a liquid network. A length-independent tension force (red) and a Brownian force (blue) combine to define junction velocities (black). (*D*) Possible rearrangements within a liquid network include junction sliding, neighbor swapping and rearrangement, loop closure and tubule spawning, growth, and fusion. (*E*) Representative simulated liquid network evolves from initially uniform honeycomb lattice to a random structure, with characteristic steady-state density. Region shown is a small segment of a much larger domain.

The tubular network of the peripheral ER (Fig. 1*A*) undergoes dynamic rearrangements (Fig. 1*B* and Movie S1) that include two frequently observed processes. First, there is the creation of new tubules, which branch from and remain connected to the existing network. Most commonly, tubule creation occurs when cytoplasmic dynein or kinesin-1 motors bind to the ER and walk along acetylated microtubules (27, 28). Other mechanisms of new tubule growth include attachment of the ER to the dynamic plus-ends of microtubules (29), or to motile organelles such as trafficking endosomes (30), lysosomes (31) and mitochondria (32). The second class of dynamic ER rearrangements arises from the inherent membrane tension in the lipid bilayer of the tubules. This tension induces junction sliding and neighbor rearrangements, akin to the T1 rearrangements observed in foams (33), leading to a net decrease in network edge length (34). As a result, loops of tubules can shrink until they vanish, often referred to as loop or ring closure. Certain loop closure events may play important functional roles in the fission of mitochondria (32) and endosomes (35), while other loop closures are rapid and do not appear to be associated with other organelles.

Similar structural rearrangements, including T1 events and ring closures, have been observed in other physical systems characterized by interfaces under an effective tension. These include foams (36, 37), microemulsions (38, 39), crystal grain growth in metals (40, 41), and patterns formed by mass-conserving reaction-diffusion systems (42, 43). The evolution of domain boundaries in these systems is driven by a curvature-dependent effective pressure and a characteristic rate of material transport across the boundary. The tendency of large domains to grow at the expense of small ones leads to domain coarsening over time with a characteristic scaling (33, 44).

Other space-tiling systems, such as epithelial cell monolayers, do not coarsen but also undergo boundary rearrangements driven by a combination of interface tension and domain pressure (45). Such monolayers exhibit T1 junction rearrangements, ring closure due to cell extrusion, and domain splitting arising from cell division. Domain splitting is also observed in ER networks, resulting from the spawning and growth of new tubules. However, the ER differs from previous classic models of foams, crystal grains, and monolayers due to the “empty” nature of the domains between tubules, with no substantial resistance expected to the exchange of cytoplasm between neighboring domains.

With the aim of connecting the small-scale dynamic rearrangements of the peripheral ER to its large-scale topological structure, we develop a simple physical model of the ER as an “active liquid network.” We first identify junction mobility and tubule spawning rate as the primary regulators of steady-state network density within the liquid network model. The balance of creation and annihilation of tubules leads to a network structure with a characteristic density and connectivity profile. Key geometric features of the ER in living cells, such as the distribution of areas between tubules and their shapes, are reproduced by this simple model. Liquid networks with physiological densities are also found to rearrange at a rate consistent with the living ER. Intriguingly, extracted laws for growth and shrinking dynamics in simulations are sufficient to recapitulate the distribution of areas, thus highlighting how large-scale structure emerges from local dynamics. Cells may modulate these properties by tuning their effective junction mobility or tubule spawning rate. This could be achieved by static tethering of the ER to the cytoskeleton and other organelles and by withdrawal of newly growing tubules in “catastrophe” events whose rate is quantified via semiautomated tracking in COS7 cells. Through computational models, analytic calculations, and quantitative image analysis of the peripheral ER in living mammalian cells, we identify physical rules governing its formation and maintenance on a cellular scale.

## Results

### Emergent Network Topology from Tension and Growth.

Inspired by the dynamic processes observed in living animal cells and prior descriptions of small ER subregions as length-minimizing networks (24–26), we build a physical model of the mammalian peripheral ER as an active liquid network. Here, we focus on two-dimensional networks, relevant for adherent mammalian cells such as COS7 cells, whose thin periphery typically accommodates only a single layer of ER tubules (46). The 2D nature of these ER networks is evidenced by the lack of degree-four junctions observed in a planar projection of the network (Fig. 1*A*). We separately explore an extension of the model to three dimensions in (*SI Appendix*, Fig. S1). The liquid network is composed of edges which transmit a membrane tension force between neighboring junctions. The membrane tension and tubule radii are assumed to be constant throughout the network.

In the low Reynolds-number environment of the cytoplasm, the motion of junction node i, located at position ${\vec{r}}_{i}$, is assumed to obey an overdamped Langevin equation (Fig. 1*C*):

[1] $$\begin{array}{c} \frac{d{\vec{r}}_{i}}{\mathrm{dt}}=-b\nabla f\left({\vec{r}}_{i}\right)+\sqrt{2D}\vec{\eta }\left(t\right). \end{array}$$

Here, b is the junction mobility (units of μm/s), $f\left({\vec{r}}_{i}\right)=\sum_{j=1}^{d}\left|{\vec{r}}_{i}-{\vec{r}}_{j}\right|$ is the total tubule length connecting junction i with its neighbors at positions ${\vec{r}}_{j}$, D is the junction diffusivity, and $\vec{\eta }\left(t\right)$ is a Gaussian distributed random noise term with zero mean and unit variance in each dimension. The first term describes the deterministic response to length-minimizing tension forces along the edges, and the second term describes a random Brownian force meant to capture the noisy environment of the cytoplasm.

With the above laws of motion, liquid network junctions are frequently pulled into close contact. Within some threshold distance of one another, junctions may swap neighbors, allowing for T1 transitions that lead to a decreasing edge length. Model results are not sensitive to the choice of threshold distance (*SI Appendix*, Fig. S2). Example neighbor rearrangements are shown in Fig. 1*D*. Occasionally, loops may form due to these neighbor swapping events, wherein two junctions are doubly connected. These loops contract over time, due to there being twice as much tension pulling them together as there is pulling them apart. The network is not allowed to rupture or fragment, a process which can be induced in vitro (47) but is not observed in our data, potentially due to the high membrane tension in the ER (26).

In addition to the tension and diffusion-driven motion of junctions, new tubule nucleation from existing tubules is modeled as an exponentially distributed random process with rate k (tubule spawning rate, units of $\mu {\text{m}}^{-1}{\text{s}}^{-1}$). Newly spawned tubules grow with a velocity v (μm/s), until coming into contact with an existing tubule, at which point the growth ceases and the tubules fuse, forming a stable junction.

In the endoplasmic reticulum, the velocity of growing tubules has been measured to be on the order of 1 μm/s (28, 48). We assume that this velocity is fast compared to the junction mobility (v≫b), so that a newly spawned tubule will span across a polygon before it substantially changes its shape. This assumption, justified via subsequently described measurements, allows us to treat tubule growth as nearly instantaneous. Additionally, the diffusive motion of junctions is assumed to be relatively small compared to experimentally observed tension-induced sliding events. With these assumptions, the behavior of the liquid network model can effectively be described by two parameters: the junction mobility, b, and the tubule spawning rate, k.

We simulate active liquid networks for a variety of parameter values, by integrating Eq. **1** forward in time (details in *SI Appendix*, *Materials and Methods*). As seen in Fig. 1*E*, an initially uniform honeycomb network develops into a random network over time. The network continuously evolves, but after a transient initial period, the tubule density remains relatively unchanged. Thus, a steady state emerges due to the competing forces of junction motion controlled by edge tension and stochastic spawning of novel tubules.

### Steady-State Density and Timescales of Liquid Networks.

**Fig. 2.**
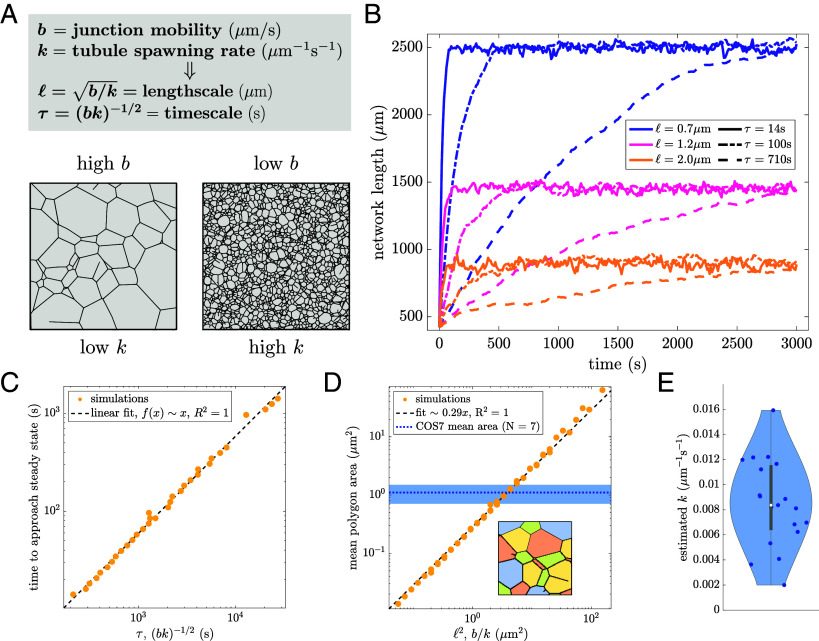
Steady-state network density and rearrangement timescales are set by two parameters. (*A*) Junction mobility and tubule spawning rate set the length and time scales (ℓ, τ respectively). (*B*) Network length over time for nine separate simulations, each starting from an initially sparse honeycomb network enclosed in a circle of radius 15 μm. The steady-state network length is set by ℓ (color) and the time to reach steady state is set by τ (line style). Other parameters are $D=10^{-5}\;\mu {\text{m}}^{2}$/s and v=1 μm/s. (*C*) Timescale to approach steady state, extracted via exponential fit of curves as in (*B*) is proportional to the intrinsic network timescale τ. (*D*) The mean area of polygons in a liquid network is proportional to $\ell^{2}=b/k$. Mean polygon area in COS7 cells is indicated by the dotted blue line; the shaded region gives intercell SD (N=7 cells). *Inset*: illustration of polygon extraction, different colored regions correspond to individual polygons. (*E*) Measured spawning rates from 19 COS7 cells, with mean and intercell SD of $8.5\pm 0.8\times 10^{-3}\;\mu {\text{m}}^{-1}{\text{s}}^{-1}$. Scatter data for (*C* and *D*) is from simulations with mobility ranging between 0.001 to 0.1 μm/s and spawning rate ranging between 0.0005 to 0.05 $\mu {\text{m}}^{-1}{\text{s}}^{-1}$.

Because the liquid network model is governed by only two parameters, dimensional analysis indicates there is a single characteristic length scale ($\ell =\sqrt{b/k}$) and timescale ($\tau =1/\sqrt{\mathrm{bk}}$) that define its behavior. Thus, changing the ratio of b and k generates networks with varying steady-state density, and changing the product of b and k affects the dynamics of the network (Fig. 2*A* and Movie S2). To illustrate this effect, the results from nine simulations of liquid networks are shown in Fig. 2*B*. Each of the nine simulations has the same initial condition but a different combination of ℓ and τ.

The total network length approaches a steady-state value over time and can be fit to an exponential of the form $f\left(x\right)=c\left(1-\exp\left(t/\tau_{\text{steady}}\right)\right)$. The parameter $\tau_{\text{steady}}$ gives an estimate of the time for liquid networks to approach steady state. This extracted time scales linearly with τ over a wide parameter range, as expected for simulations with a single dominant timescale (Fig. 2*C*).

To measure steady-state density one can consider the area of polygons (minimal loops) within the network. The mean area of these polygons scales linearly with $\ell^{2}=b/k$ (Fig. 2*D*). It is therefore possible to construct networks with the same density as observed in living cells simply by tuning ℓ. From a linear fit of the mean area across a range of b and k values, we obtain ⟨A⟩≈0.29b/k.

The overall steady-state density of liquid networks can be captured by a simple equation for the total network length as a function of the new tubule spawning rate k and mobility b. For simplicity, we consider a network of hexagonal polygons. The total network length L can either grow via spawning of new tubules across the polygons, or decrease due to junction sliding:

[2] $$\begin{array}{c} \frac{\mathrm{dL}}{\mathrm{dt}}=kL\lambda \sqrt{3}-\gamma bn. \end{array}$$

Here, λ is the average edge length, $\sqrt{3}\lambda$ is the distance between parallel edges of a hexagon, n is the total number of junctions in the network, and γb describes the average sliding speed of a junction. At each individual junction, the value of γ must be between 0 and 3 depending on the angles between the adjacent tubules. The speed of growing tips is assumed to be sufficiently fast that growth events are instantaneous and there is no effect from the diffusion of junctions.

The total network length can be expressed in terms of the average edge length (λ) and the number of junctions (n) using the fact that the number of edges (e) in a hexagonal lattice is $e=\frac{3}{2}n$. Thus, $L=\frac{3}{2}\lambda n$ and at steady state $\frac{\mathrm{dL}}{\mathrm{dt}}=0$ gives an average area of:

[3] $$\begin{array}{c} \left\langle A\right\rangle =\frac{3\sqrt{3}}{2}\lambda^{2}=\gamma \frac{b}{k}. \end{array}$$

This predicted scaling of mean area with b/k aligns with expectations from dimensional analysis and agrees with the fit of simulation results. Notably, an analogous scaling law relating network dynamics and the average edge length is also observed for three-dimensional liquid network simulations (*SI Appendix*, Fig. S1). However, such 3D networks also depend on an additional length scale (the contact radius within which a growing tube is capable of fusing into the network), which modulates network pore size and density.

The new tubule spawning rate k can be extracted directly from observations of ER dynamics in live COS7 cells (Fig. 2*E* and details in *SI Appendix*, *Materials and Methods*). Together with the measured polygon area, this allows the effective mobility of the ER junctions in COS7 cells to be estimated as b=⟨A⟩k/γ=0.03μm/s. We note that the relevant mobility is indeed much slower than the tubule growth speed (b≪v), justifying our assumption of a single dominant length and time scale. One advantage of this approach is that tubule spawning rate is simple to directly measure from experimental data whereas junction mobility is a more opaque quantity. Thus, it is possible to calculate ER junction mobility via mean polygon area and spawning-rate measurements by taking advantage of the steady-state properties of liquid networks.

### Scale-Invariant Network Structure Reproduces ER Morphology.

**Fig. 3.**
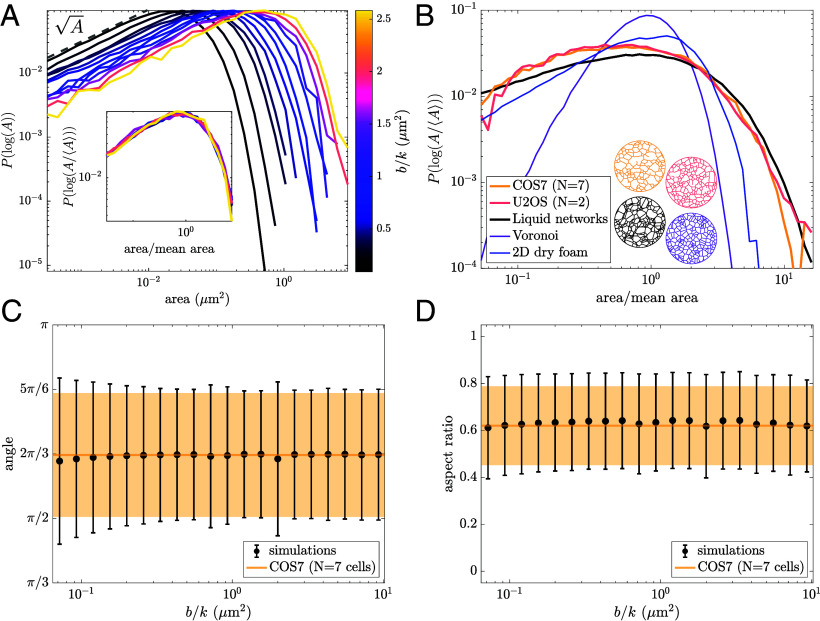
Scale-invariant liquid network model reproduces peripheral ER morphology. (*A*) Distribution of polygon areas in simulated liquid networks for a range of b/k values. Bin sizes are logarithmic. *Inset*: distributions collapse onto a single curve when normalized by mean area, revealing scale-invariant behavior of liquid networks. (*B*) Distribution of normalized polygon areas from the ER networks of COS7 and U2OS cells (yellow and pink, respectively) exhibit similar scaling to simulated liquid networks (black). For comparison, the distribution of areas for Voronoi networks and experimental measurements of a two-dimensional dry foam (data from ref. 49) are also shown (purple and blue lines). Example networks in matching color *Below*. (*C*) Mean and SD of angles between 3-way junctions in liquid networks (black) and the COS7 ER (yellow). (*D*) Mean and SD of aspect ratio (shortest dimension/longest dimension) in liquid networks (black) and the COS7 ER (yellow).

While the two parameters of the liquid network model set length and time scales, any dimensionless metric of network structure must be parameter-independent. In particular, we consider the full distribution of polygon areas for a range of ℓ values (Fig. 3*A*). As the ratio of mobility to spawning rate increases, the distributions shift to the right, indicating an increase in large areas and a decrease in small areas. However, the overall shape of the distribution remains unchanged. At low areas, the log-binned distribution scales as $∼\sqrt{A}$, and at high areas, it decays exponentially. When normalized by the mean area, the distributions collapse onto a single universal curve (Fig. 3*A*, *Inset*), highlighting the scale-free nature of the model. Thus, increasing mobility is equivalent to “zooming in” on a patch of network, while increasing spawning rates leads to a denser network, or “zooming out.”

Notably, the parameter-independent shape of the polygon area distribution for liquid networks provides a good approximation to that of the ER in living cells. In Fig. 3*B*, area distributions extracted from two different cell types commonly used to study the peripheral ER are shown (details in *SI Appendix*, *Materials and Methods*). Both COS7 and U2OS cells (monkey kidney and human osteosarcoma, respectively) exhibit remarkably similar scaling, collapsing onto a single curve. The exponential drop-off at large areas is clearly conserved across experiments and simulations. The experimental measurements exhibit a slight enhancement of small-area polygons as compared to liquid network simulations. However, limitations in imaging resolution and segmentation prohibit the extraction of a reliable power-law for small-area scaling in the distribution.

The distributions for two other families of network are provided in order to demonstrate that the close match to ER morphology is not exhibited by other commonly studied 2D networks. The area distribution of simulated Voronoi networks (purple line) is comparatively narrow and sharply peaked around the mean. Experimental imaging data of a two-dimensional dry foam (49) (blue line), shows that the area distribution of foams is broader than Voronoi networks but still narrower than the ER and liquid networks. The foam also exhibits an exponential drop-off at large areas but has a linear scaling in the logarithmic distribution of small-area polygons. We note that extending the liquid network model to three dimensions yields a distinctly different scaling for the size distribution of small pores, akin to that observed in foams and random-line networks (*SI Appendix*, Fig. S1).

For 2D liquid networks, it is possible to estimate the functional form of the polygon area distribution (black line) using only the rules for polygon growth and splitting, as described in the following section.

Other metrics of shape further confirm the similarities between the liquid network model and experimental ER networks. The distribution of angles between neighboring 3-way junctions is centered at 2π/3 or $120^{{}^{\circ}}$. This is a universal property of two-dimensional networks composed of degree 3 junctions. The SD of angles at 3-way junctions is also shown to be similar for simulated and observed ER networks across a wide range of parameter values (Fig. 3*C*). Finally, the aspect ratio of polygons (ratio of shortest dimension to longest dimension) is calculated in both simulated and experimental networks. This provides another dimensionless, scale-free measure of shape: The mean and SD of polygon aspect ratio are constant over a wide range of parameter values and approximately match experimental measurements (Fig. 3*D*). Notably, the realistic variances of the aspect ratio and junction angles arise from heterogeneity in polygon shapes and are not related to the Brownian forces on the junctions (*SI Appendix*, Fig. S2 *A*–*C*), which are taken to be very small in the simulations.

### Model Dynamics Determine Network Rearrangement Rates.

Having demonstrated that the liquid network model approximately matches the steady-state morphology of ER networks, we next proceed to compare the dynamics of simulated and observed network structures. We consider two metrics for the dynamic rearrangement of the networks, demonstrating how parameters extracted from measuring polygon size and new tubule spawning enable accurate prediction of the time-evolution of ER network structure.

#### Edge mean minimal distance.

**Fig. 4.**
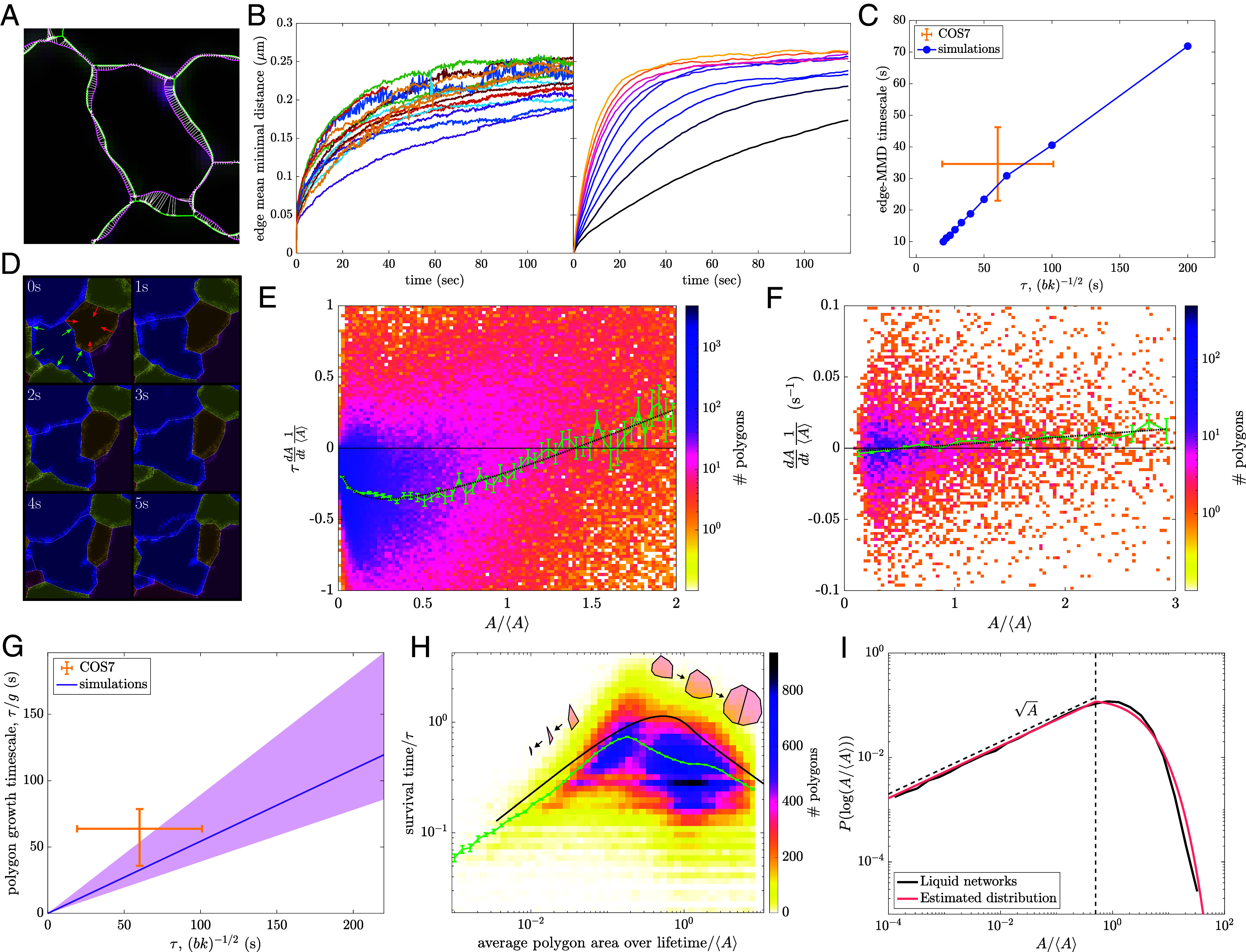
Dynamics of liquid networks predict ER rearrangement timescales and give rise to emergent polygon area distribution. (*A*) Confocal images of COS7 ER with 1 s interval between first and last frames (green, pink). Extracted networks overlaid in matching colors. White arrows indicate minimal distance from meshed points on final network (pink) to meshed points on the initial network (green). (*B*) *Left* panel: edge mean minimal distance (EMMD) over time for 16 peripheral ER networks of COS7 cells. *Right* panel: EMMD of liquid networks with $\tau ={\left(bk\right)}^{-1/2}$ varying across one order of magnitude and fixed $\sqrt{b/k}=2$ μm. (*C*) Exponential timescale for EMMD to approach steady-state scales with τ for liquid network simulations. Yellow lines show mean value and intercellular SD for COS7 cells. (*D*) Example polygon tracking of experimental data to quantify polygon growth and shrinking rates. Each frame is 5.0×5.0 μm with a timestep of 2 s. Red and green arrows indicate shrinking and growing polygons, respectively. (*E*) Nondimensionalized growth rates normalized by mean area and network timescale for 10 liquid network simulations. The green curve indicates mean (and SE of the mean) within coarse bins of normalized area. Dotted black curve indicates fit to Eq. **4**, giving β=0.78,g=1.69,andh=1.85. (*F*) Mean growth rates normalized by mean area and their dependence on relative polygon size from 7 experimental COS7 peripheral ER networks. The green curve indicates mean (and SE of the mean). Dotted black curve indicates fit to Eq. **4**, with β fixed at 0.78 giving $g/\tau =0.018\;{\text{s}}^{-1}$ and $h/\tau =0.015\;{\text{s}}^{-1}$. (*G*) Polygon growth timescale (defined as τ/g) from 7 COS7 cells agrees with the simulated timescales extracted from simulations. (*H*) Polygon survival times normalized by τ for 10 liquid network simulations. The green curve indicates mean (and SE of the mean) within bins of area. The black curve indicates theoretical prediction. *Inset*: representative trajectories for shrinking and splitting polygons. (*I*) Distribution of normalized polygon areas in a simulated liquid network (black) compared to analytically approximated area distribution (pink). The vertical dashed line shows $A^{*}$, where small-area and large-area solutions are joined.

To quantify the motion of individual tubules over time and the resulting changes in network structure, the edge mean minimal distance (EMMD) is calculated between the first and all subsequent networks, as described in *SI Appendix*, *Materials and Methods*. Briefly, the network is meshed and for each point on subsequent networks, the minimal distance to a point on the starting network is found; these minimal distances are averaged to find the EMMD. The EMMD grows over time (Fig. 4*B*, *Left* panel), with an initial jump between the first and second frames that can be attributed to localization error and network segmentation artifacts. The same calculation is performed for simulated liquid networks with a matching average polygon size and a range of timescales.

Exponential fits of the EMMD growth over time give an effective timescale for the rearrangement of the network. As expected, this rearrangement time scales linearly with the intrinsic timescale τ of the simulations (Fig. 4*C*). The observed rearrangement timescale for the COS7 ER is 35±11 s, implying that during this time edges are significantly displaced from their original location. This timescale is of similar magnitude to the time required for the ER as a whole to explore a large fraction of the cytoplasm (50). Notably, this rearrangement timescale is consistent with the simulation model, given the parameters b and k extracted from measurements of polygon area and tubule spawning rate. Thus, the liquid network model makes it possible to connect two seemingly independent dynamic processes—new tubule growth and rearrangement of existing edges.

#### Polygon growth rates.

An additional metric to quantify the dynamic behavior of liquid networks arises from considering the growth and shrinking rates of polygons (independent of splitting events due to new tubule growth). Polygons are tracked in time and space using conventional particle tracking software (51) (details in *SI Appendix*, *Materials and Methods*). We calculate the normalized growth rate for all tracked polygons in several COS7 ER networks and simulated liquid networks. The normalized growth rate is defined to be the time-derivative of each polygon’s area scaled by the average polygon area in that cell. For simulated networks, the growth rate is rescaled by the characteristic model timescale $\tau ={\left(bk\right)}^{-1/2}$, allowing simulations with different parameter values to be analyzed together. By determining the relationship between growth rate and polygon area the underlying laws governing the dynamics of the network can be probed.

A characteristic behavior of liquid networks is the growth of large polygons and the shrinking of small ones (Fig. 4*E*). This is similar to von Neumann’s law for foams, which dictates that polygons with more than 6 sides grow while those with fewer sides shrink (36). In liquid networks, as in foams (33) and crystal grain boundaries (41), polygons that are larger than average tend to have more sides, as a greater number of average-sized neighbors can fit around them (*SI Appendix*, Fig. S3*A*). Consequently, such above-average polygons tend to have internal angles greater than $120^{{}^{\circ}}$, causing them to grow under tension. As noted in *SI Appendix*, the growth and shrinking rates for a regular n-gon are expected to be proportional to its perimeter, which scales roughly as $\sqrt{A}$. For large polygons, the number of neighbors should also increase as the polygon grows, giving a steeper dependence of the growth rate on area. We fit the average simulated growth rates to the following expression: (Fig. 4*E*):

[4] $$\begin{array}{c} \tau \frac{1}{\left\langle A\right\rangle }\frac{\mathrm{dA}}{\mathrm{dt}}=g{\left(\frac{A}{\left\langle A\right\rangle }\right)}^{\beta }-h\sqrt{\frac{A}{\left\langle A\right\rangle }}, \end{array}$$

where the prefactor g encapsulates the rate of large polygon growth and h describes the rate of small-polygon shrinkage. We note that the above equation constitutes an approximate ansatz that describes the distinct dynamic behavior of polygons at the extremes of the area distribution. From the fitted function, we can extract the typical rate $k_{\text{grow}}=g/\tau$ for the growth of large polygons.

When tracking polygons in images of live ER, the average growth rate is also negative for small polygons and positive for large ones, as in the liquid network model. Due to the limited data at small polygon areas, we fix the value β=0.78 as fitted for simulated networks, and fit the remaining coefficients in Eq. **4** to the experimental data (Fig. 4*F*). This enables the extraction of a growth rate for large polygons, in real time units. As shown in Fig. 4*G*, the estimated growth rate for COS7 ER falls within range of the predicted value for liquid networks with the appropriate timescale τ. Thus, by measuring the average polygon area and rate of new tubule spawning (to set parameters b,k), the liquid network model makes it possible to predict the typical growth rate of large polygons in the ER network, thereby connecting distinct dynamic processes.

These results demonstrate that liquid networks are not only able to replicate key steady-state structural features but also capture the rearrangement timescales of living ER networks, providing a connection between morphology and tubule dynamics.

### Steady-State Structure Emerges from Polygon Dynamics.

The dynamic behavior of network polygons can be abstracted still further by considering them as a population of individual aspatial entities capable of growing, shrinking, and splitting (Movie S3). The drift velocity of polygon areas v(A)=dA/dt is set by Eq. **4**. The rate of splitting is expected to be proportional to the perimeter of a polygon, which scales as the square root of the area: $k_{\text{split}}=\hat{k}\sqrt{A}$. This simplified description neglects noise in the polygon trajectories, assuming the dynamics of each is consistent with the average behavior of the population.

#### Polygon survival times.

The normalized area of a given polygon [$\hat{A}\left(t\right)=A\left(t\right)/\left\langle A\right\rangle$] can be treated as a deterministically growing or shrinking quantity, computed through integration of Eq. **4**. The polygon will grow if its initial normalized area is above the cutoff value of ${\hat{A}}_{c}={\left(h/g\right)}^{1/(\beta -1/2)}$ and will shrink otherwise. The polygon trajectory is terminated by a stochastic splitting event with rate $k_{\text{split}}$.

For a shrinking polygon with initial area ${\hat{A}}_{0}$, we can compute the time to closure as $t^{*}=\int_{0}^{{\hat{A}}_{0}}\frac{-d\hat{A}}{v(\hat{A})}$. The survival probability S(t) to time $t<t^{*}$ is given by

[5] $$\begin{array}{c} S\left(t\right)=e^{-\hat{k}\sqrt{\left\langle A\right\rangle }\int_{0}^{t}\sqrt{\hat{A}\left(t^{\prime }\right)}dt^{\prime }}. \end{array}$$

and the average survival time can be found as

[6] $$\begin{array}{c} \tau =t^{*}S\left(t^{*}\right)-\int_{0}^{t^{*}}t^{\prime }\partial_{t^{\prime }}S\left(t^{\prime }\right)dt^{\prime }=\int_{0}^{t^{*}}S\left(t^{\prime }\right)dt^{\prime }. \end{array}$$

For growing polygons, an analogous expression for the average survival time is given by replacing $t^{*}\to \infty$. The resulting estimate for the survival time approximately matches the behavior of polygons in liquid network simulations (Fig. 4*H*). Notably, polygons with intermediate areas have the longest survival time because they are both slow to shrink and also have a low splitting rate.

Because this simplified model of polygon dynamics does not incorporate stochastic noise in the trajectories, the approximated survival times tend to be slightly overpredicted. In simulations, some polygons with area below ${\hat{A}}_{c}$ may nevertheless be growing and some larger polygons may be shrinking. As a result, polygons spend less time trapped in the intermediate-area state where deterministic dynamics are very slow; they are more likely to fluctuate to a more extreme small or large area that quickly leads to a shrinkage or a splitting event, thereby reducing the survival time.

#### Steady-state area distribution.

Making the same approximation of deterministic dynamics for the population of polygon areas, we define P(A) as the area distribution function, whose steady-state form must encompass a balance between small polygons disappearing due to shrinking and large polygons splitting into new ones.

In the limit of very small areas, the formation and disappearance of polygons due to splitting is negligible compared to the flux associated with polygon shrinking (*SI Appendix*). The overall rate of polygons disappearing when their area shrinks to zero can be estimated as

[7] $$\begin{array}{c} J_{0}=\lim_{A\to 0}v\left(A\right)P\left(A\right)=\lim_{A\to 0}\frac{h}{\tau }{\left\langle A\right\rangle }^{1/2}\sqrt{A}P\left(A\right). \end{array}$$

Because this flux must be a finite nonzero value, the small-area limit of the distribution is set to $P\left(A\right)\to c_{1}/\sqrt{A}$, where $c_{1}$ is a constant. A logarithmic transform gives the scaling $P\left(\log\;A\right)∼\sqrt{A}$, as observed in Fig. 3*A*.

In the limit of very large polygons, the distribution evolves due to area growth and splitting, and the steady-state form can be found by solving the resulting equation:

[8] $$\begin{array}{c} \frac{\partial P}{\partial t}=-\frac{\partial }{\partial A}\left[v(A)P\right]-\hat{k}\sqrt{A}P=0 \end{array}$$

[9] $$\begin{array}{c} P\left(A\right)\to c_{3}c_{1}A^{-\beta }e^{-c_{2}A^{z}}, \end{array}$$

where $z=\frac{3}{2}-\beta$, $c_{2}=-\frac{\hat{k}{\left\langle A\right\rangle }^{\beta -1}}{\mathrm{gz}}$, and $c_{3}$ is a constant.

In order to construct a full approximate distribution, the two limits for small and large areas are married together at some intermediate value $A^{*}$, thereby enforcing a value for the coefficient $c_{3}={\left(A^{*}\right)}^{1-z}\exp\left(c_{2}A^{*z}\right)$. The coefficient $c_{1}$ is obtained by normalizing P(A). Furthermore, the total rate at which polygons disappear ($J_{0}$) and the rate at which new ones are produced ($J_{\text{split}}=\int_{0}^{\infty }\hat{k}\sqrt{A}P\left(A\right)dA$) must be equal at steady state. This constraint fixes the transition value $A^{*}\approx 0.12b/k$.

These calculations lead to a predicted average polygon area ⟨A⟩≈0.23(b/k), similar to the fitted relationship in Fig. 2*D*. Furthermore, the overall distribution of polygon areas, with a polynomial scaling at small A and an exponential scaling at large A, approximately reproduces the observed distribution from liquid network simulations (Fig. 4*I*). We note that the only fitting parameters employed in this analysis are the values of g,h,β in the expression for polygon growth rates as a function of area (Eq. **4**). Thus measurement of polygon dynamics can be leveraged to approximately predict the steady-state architecture of the liquid network as well as the ER network structure in live cells.

### Pinning to Static Structures Increases Network Density.

We have shown how the structure and dynamics of liquid networks are governed by two parameters: the junction mobility and tubule spawning rate. In the next two sections, we explore how the cell effectively controls these parameters to modulate ER properties, first examining how junction mobility can be tuned by tethering of the ER to static structures.

The ER exists within the crowded, complex environment of the cytoplasm. It is pinned to the cytoskeleton via contacts with microtubules and actin filaments (52, 53). The ER also forms critical contact sites with mitochondria (28, 32, 54), the plasma membrane (55), the Golgi (56, 57), endosomes (30, 35) and other organelles (1). Quantification of ER network dynamics in plant cells has indicated that certain points along the network remain persistent over minute-long timescales (24). To determine the effect of connections to static structures, we introduce a process for temporarily immobilizing junctions in liquid networks via pinning (rate $k_{p}$). An unpinning process (rate $k_{u}$) allows for a steady-state number of pins (Movie S4). The ratio $n_{p}=k_{p}/k_{u}$ sets the average number of pins in the model. We analyze systems with variable pin densities and with a wide range of pin persistence times.

**Fig. 5.**
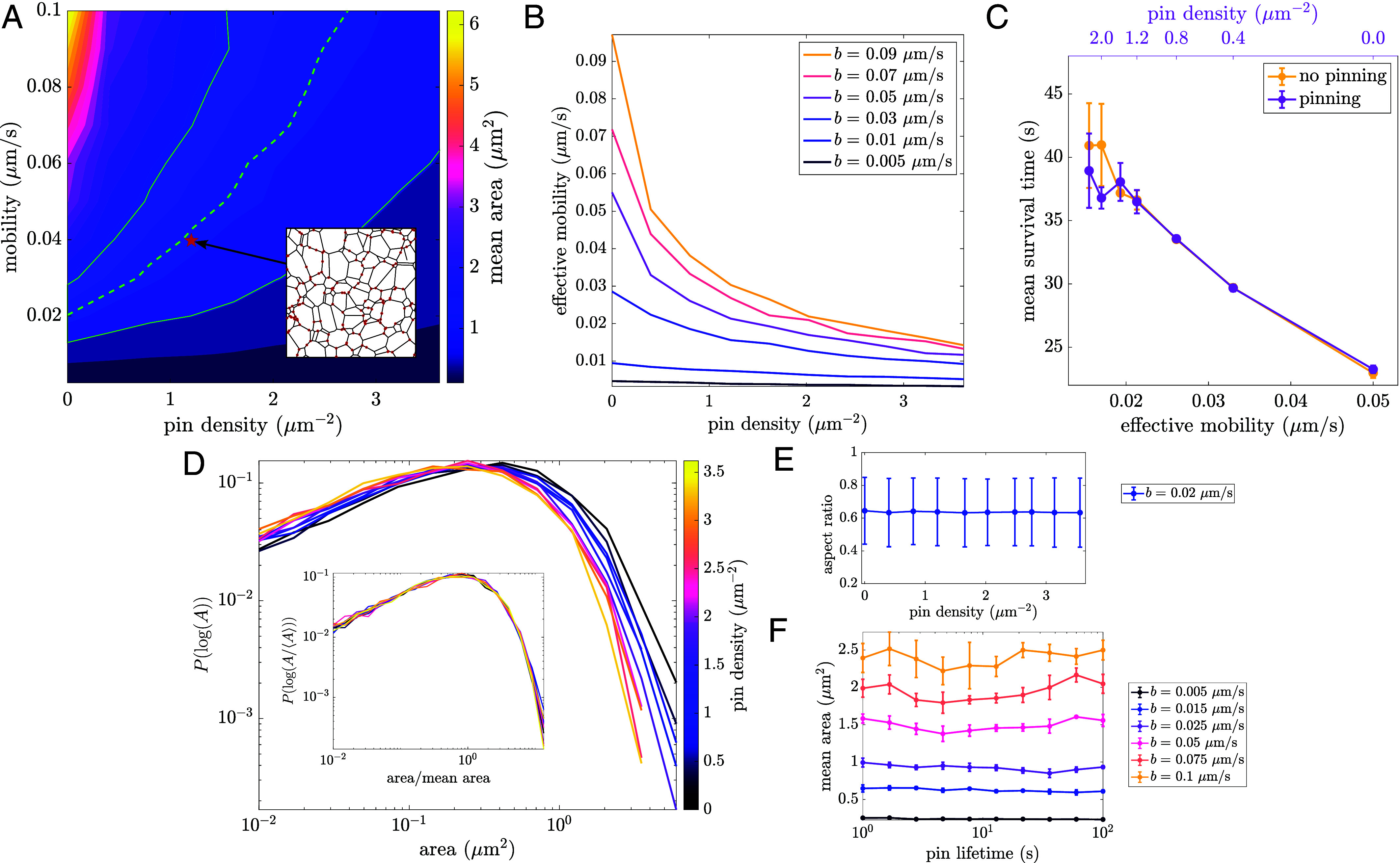
Tethering to static structures reduces effective mobility and increases network density. (*A*) Dependence of mean area on mobility and pin density. The dashed green line indicates contour corresponding to mean COS7 area, with the shaded green region indicating SD across all cells (N=7). *Inset*: depicts an example 10×10μmsnapshot of a liquid network with pinned nodes highlighted in red. (*B*) Effective mobility, defined as $b_{\text{eff}}=\left\langle A\right\rangle k/\gamma$, as a function of pin density for different mobilities. (*C*) Mean polygon survival time for simulations with and without pinning. Yellow curve: no pinning, varying junction mobility b (*Bottom* axis). Pink curve: different pin densities (*Top* axis), with constant mobility b=0.02 μm/s, plotted against the effective junction mobility $b_{\text{eff}}$ (*Bottom* axis). Matching effective mobilities gives similar results with and without pinning. Error bars indicate SD across three simulations for each data point. (*D*) Reversible pinning leads to denser networks, shifting the area distributions. *Inset*: normalized area distributions collapse to a single curve for all pin densities. (*E*) Aspect ratio is unchanged across a range of pin densities. (*F*) Mean area remains constant across a wide range of pin lifetimes. All results are with a tubule spawning rate of $k=0.005\;\mu {\text{m}}^{-1}{\text{s}}^{-1}$.

To begin, we find how mean area depends on both junction mobility and pin density, calculated as $n_{p}$ divided by total simulation area (Fig. 5*A*). For a fixed mobility, increasing the density of pins leads to a denser network with smaller mean areas. This effect is most pronounced for larger mobilities, leading to a steep decrease in mean area as pin density increases. The contour corresponding to the average area across COS7 cells is shown in green, indicating a wide range of possible mobility and pin density combinations in experimental networks.

The overall effect of pinning is to reduce the rate at which the network can relax and rearrange. For any given pin density, we can define an “effective mobility” ($b_{\text{eff}}=\left\langle A\right\rangle k/\gamma$, with γ=0.29) by finding the value of b that would give the same average polygon area in the absence of pinned points. At low pin density, $b_{\text{eff}}\approx b$, but as the pin density grows, the effective mobility steeply decreases (Fig. 5*B*) as the rearrangement of the network is slowed down. The effect of pinning on the increasing survival time of individual polygons is encompassed by the accompanying slow-down in effective mobility (Fig. 5*C*).

In liquid networks, pinning to static structures or other organelles may thus limit network mobility and increase the corresponding density of ER network tubules in critical regions of the cell. For instance, this mechanism could aid the coalescence of ER around mitochondria contact sites.

Beyond tuning the density of the network, pinning has little effect on steady-state properties. Area distributions shift as a function of pin density, but when normalized by mean area, the data collapse onto a single curve (Fig. 5*D*) just as before (Fig. 3*A*). There is also no effect on the mean and variance of polygon shape, as measured by aspect ratio (Fig. 5*E*). Furthermore, altering the pinning and unpinning rates (thus probing a wide range of pin lifetimes while maintaining a fixed pin density) has no effect on the steady-state structure of liquid networks (Fig. 5*F*).

### Tracking Tubule Spawning in COS7 ER Reveals Rate of Catastrophe.

Another mechanism through which cells can modulate ER network properties is by tuning tubule spawning rate. Within living cells, newly spawned tubules often cease growth and retract, a process we refer to as “catastrophe.” Independent of the underlying mechanism of growth [e.g. ER sliding or TAC events (27–29)], we quantify ER tubule catastrophe rates in COS7 cells, and explore the effect of catastrophe on network structure.

**Fig. 6.**
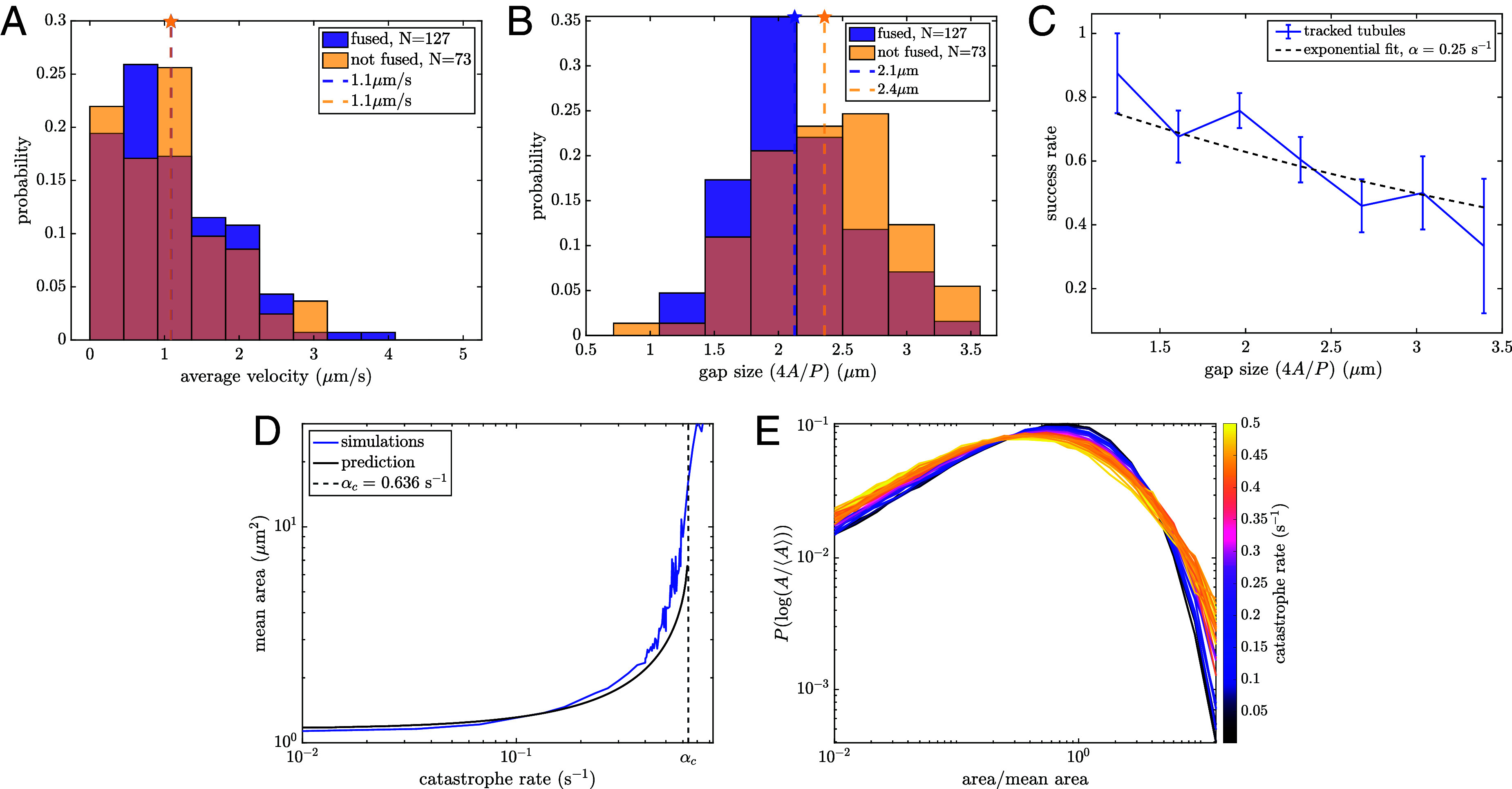
Growing tubules undergo catastrophe events that alter polygon size distributions. (*A*) Velocity distributions of growing tubules that lead to both successful and unsuccessful fusion. Mean of each distribution indicated by a dashed line. (*B*) Distribution of gap sizes across which tubules are growing for both successful and unsuccessful fusion events. Mean of each distribution indicated by a dashed line. P=0.001 for a one-sided Student’s *t* test. (*C*) Success rate as a function of gap size d, with fit to $S\left(d\right)=e^{-\alpha d/v}$ indicated by a dashed line. (*D*) Mean area grows as a function of catastrophe rate. The black line marks analytic prediction. The dashed vertical line shows predicted critical rate beyond which mean areas diverge. (*E*) Normalized area distributions for liquid networks with increasing catastrophe rates. High catastrophe rate leads to an enrichment of both small and large areas.

The tips of newly spawned tubules are tracked within COS7 cells (details in *SI Appendix*, *Materials and Methods*). In addition to positional data, it is also recorded whether the growth is successful in fusing with a neighboring tubule (fused) or if it ultimately retracts (not fused). Using the position of tracked tips, the average velocity until fusion or until the time of retraction is calculated. The distribution of growth speeds is broad (Fig. 6*A*), with an average and SD of 1.09±0.75 μm/s, consistent with previous measurements (28, 48). No significant differences in the distribution are observed between tubules that successfully fuse and those that do not.

We next calculate the gap size that growing tubules must traverse in order to fuse, a quantity which differs between successful and unsuccessful spawning events (Fig. 6*B*). Here, gap size is the approximate diameter of the enclosing polygon (d=4A/P, with A area and P perimeter, equal to the inscribed diameter of regular polygons). On average, successful growth events traverse shorter gap sizes (2.1±0.5 μm) than unsuccessful events (2.4±0.5 μm). This effect can also be visualized by considering the success rate of newly spawned tubules, which decreases as a function of gap size (Fig. 6*C*).

An estimate for the catastrophe rate in living ER networks can be extracted by fitting the experimental success rate to $S\left(d\right)=e^{-\alpha d/v}$ (Fig. 6*C*). Here, d is the gap size, v=1.1 μm/s is the average velocity of all measured growth events, and α is the catastrophe rate and fit parameter. This gives $\alpha_{\text{ER}}=0.25\pm 0.04\;s^{-1}$.

To explore how these events affect the steady-state structure of the network, we introduce a constant-rate (Poissonian) catastrophe process for each growing tubule in the liquid network simulations. A single parameter (α) controls the rate at which a growing tip ceases forward motion and begins to retract due to membrane tension. Simulations are performed with experimentally relevant choices for mobility and spawning rate (b=0.02 μm/s, $k=0.005\;\mu {\text{m}}^{-1}{\text{s}}^{-1}$) across a wide range of catastrophe rates, and the steady-state structural properties of the network are analyzed.

At small catastrophe rates, the mean area of polygons in the network remains relatively unchanged from the case with no catastrophe (Fig. 6*D*). As the catastrophe rate increases, it becomes more likely that growing tubules will retract before fusing, especially across larger gaps. Thus, there are fewer splitting events of large areas, leading to an increase in mean area. The larger average gap size, in turn, leads to even fewer fusion events. As the catastrophe rate grows sufficiently large, the persistence length of growth events becomes smaller than the average gap they must traverse. The mean area is then expected to diverge beyond a critical catastrophe rate, as confirmed by simulations (Fig. 6*D*).

The average effect of catastrophe on steady-state structure in liquid networks can be analytically approximated by modifying Eq. **2** for the total network length to incorporate the success rate S(d) of new growth events:

[10] $$\begin{array}{c} \frac{\mathrm{dL}}{\mathrm{dt}}=kL\lambda \sqrt{3}e^{-\sqrt{3}\alpha \lambda /v}-\gamma bn. \end{array}$$

At steady state, this reduces to the following transcendental equation for λ,

[11] $$\begin{array}{c} \lambda^{2}e^{-\sqrt{3}\alpha \lambda /v}=\frac{2\gamma b}{3\sqrt{3}k}. \end{array}$$

The derivative of λ with respect to α approaches infinity at $\alpha_{c}=\frac{1}{e}{\left(2\sqrt{3}k/\gamma b\right)}^{1/2}$, indicating that no finite solution is possible above this critical catastrophe rate. For the simulation parameters used in Fig. 6*D*, the critical value is given by $\alpha_{c}=0.64\;{\text{s}}^{-1}$. Solving for λ numerically, the polygon mean area (Eq. **3**) can be found as a function of catastrophe (black curve in Fig. 6*D*). The divergence of average area with increasing catastrophe rate is successfully predicted by this analytic model. Using this model for catastrophe (*SI Appendix*, Fig. S4), we additionally demonstrate that liquid networks can form quasi-stable architectures without a boundary or tethering to static structures, so long as there is sufficient growth to counteract the tension-induced shrinking.

Notably, the catastrophe process affects not only the average polygon area, but also the normalized distribution of areas (Fig. 6*E*). The altered distributions arise because catastrophes have a greater effect on large than on small polygons. Thus, normalized distributions for systems with frequent catastrophes have a fatter tail of large-area polygons that are unlikely to be split by a successful new tube fusion.

## Discussion

Physical modeling of the peripheral ER as an active liquid network elucidates a fundamental connection between subcellular dynamics and organelle structure. The behavior of liquid networks is effectively described by two main parameters: the junction mobility and tubule spawning rate. A characteristic network density and connectivity emerges from a balance between tubule creation and the contraction of small polygons. This model reproduces key geometric features of the peripheral ER in adherent mammalian cells, such as the typical shape and distribution of areas between tubules. We find that liquid networks are able to replicate physiological rearrangement timescales. Quantifying polygon dynamics in these systems allows us to derive the distribution of areas, thus forming a connection between emergent steady-state structure and the underlying dynamics. Finally, by considering the effects of static tethering points and catastrophe of tubule growth we explore how the cell can alter the effective junction mobility and tubule spawning rate to modulate network properties.

In this work, the complex protein-studded membrane structure of the peripheral ER is reduced to a spatial graph of junctions connected by one-dimensional, constant-tension, fluid-like edges. This simplified model is able to recapitulate many of the structural and dynamic properties of the living ER, while remaining agnostic to the specific details of membrane-shaping and dynamics at the nanometer scale. In reality, the effective tension driving the shortening of ER tubules could be modulated by varying tubule radii and by the distribution of tubule-stabilizing proteins such as the reticulons (58). Both tubule radii and reticulon distributions are spatially heterogeneous across the network (14), potentially giving rise to tension gradients. Fluctuations in tension may also arise from cis-dimerization of membrane proteins such as atlastin (26). More complicated ER structures, such as fenestrated sheets (46), may also influence the tension and hence the dynamics of the surrounding network. Furthermore, tubule spawning rate may be heterogeneous due to varying distributions of microtubules and motors. These effects may account for some of the spatial variability in ER density observed in cells (59).

An additional simplifying assumption inherent to the liquid network model is the ability of individual tubules to straighten on a timescale faster than the node rearrangements. While some bent tubules can be seen in the peripheral ER, their persistence length (22) is usually much longer than the typical polygon size. Thus the kinks that sometimes appear in long ER tubules are likely associated with connections to other cellular structures (analogous to the “pinning points” discussed above). The constant, isotropic mobility coefficient for network nodes is another simplification, neglecting the differences in friction for dragging a tubule perpendicular to its axis, versus sliding a junction along the tubule membrane. Despite these simplifications, the liquid network model captures many aspects of ER structure and dynamics, linking local rearrangements to the network’s large-scale architecture.

The model presented here is most relevant for two-dimensional networks such as those observed in the periphery of COS7 cells. In many other cell types, the ER forms three-dimensional structures. Such networks require an additional length scale defined by the range within which a growing tubule tip can fuse into another tubule. This length scale alters the dependence of network density on the kinetic parameters b,k. While we outline scaling behaviors of 3D liquid networks in *SI Appendix*, more detailed exploration is left for future work. Further high-resolution imaging and quantification of 3D ER network architecture will also be necessary to probe the validity of the model in 3D.

Alternative 3D models can also give rise to network-like structures. In particular, microemulsions of lipids, water, and surfactants can form bicontinuous cubic phases whose density is governed by the relative concentrations of the components (38, 39). Such structures coexist with lamellar phases, with the balance modulated by the curvature preferences of the lipid layers (39). These models may be particularly relevant for understanding the transition of the ER between network-like and sheet-like structures, which can be tuned by changing the distribution of preferred membrane curvatures (19). While our liquid network model focuses on structures emerging through tension and growth, future work comparing the resultant dynamics and architecture to networks arising via phase transition may prove informative. In particular, such connections may shed light on in vitro observations of ER network formation in the absence of cytoskeletal filaments needed for new tubule extension (60).

Maintaining an organelle as an active, dynamic liquid network incurs an energy cost for the cell. In particular, motor activity or microtubule polymerization is required to grow new tubules that split polygons and interrupt coarsening. Given that individual kinesin motors burn one ATP per 8-nm step (61), we would expect the rate of energy consumption associated with network maintenance to scale as $\sqrt{2\gamma /\sqrt{3}}Lk\ell /ds$, where L is total network length, $\ell =\sqrt{b/k}$, ds is the motor step size, and γ is the scaling factor relating average polygon area to $\ell^{2}$. For the cells considered here, this would amount to roughly $2\times 10^{3}$ ATP/s. Notably, this maintenance cost is orders of magnitude lower than the estimated energy consumption (∼10^8^ ATP/s) associated with synthesizing the proteins shipped from the ER in the secretory pathway (62–64).

For this modest energetic cost, the dynamic network of the ER provides the cell with a number of functional benefits. As a topologically isolated space with high calcium concentration, the ER provides a compartment for the efficient folding of proteins destined for the extracellular environment (7, 8). Its dense network structure enables the rapid and proximal delivery of calcium ions into the cytoplasm (6) during localized signaling events known as puffs or sparks (65). The well-connected network architecture also allows for rapid search by newly folded proteins to encounter exit sites in the ER (59). Furthermore, the density of the network makes it possible for the ER to form a plethora of contact sites for the transport of proteins, lipids, and ions to and from other organelles such as mitochondria and endo/lysosomes (31, 32, 35, 66).

The dynamic rearrangements of the network could allow for rapid structural response of the ER to local and global perturbations. For example, the increased network density associated with pinning points may aid the accumulation of ER tubules near mitochondrial contact sites, where the ER is known to play an important role in mitochondrial fission and fusion (32, 67). Network dynamics could also allow the ER to restructure around rearranging organelles or in response to cytoplasmic deformation in motile cells (68).

The interplay between microtubules and peripheral ER (69) enables the network to maintain its structure against tension. Suppression of motor proteins that drive tubule extension results in retraction of the ER network from the cell periphery (70). In addition, depolymerization of microtubules following nocodazole treatment leads to the withdrawal of peripheral ER tubules and an increase in perinuclear ER sheets and cisternae (71). By maintaining the ER in a state of constant tension, poised on the verge of retraction and controlled by driven extension along the cytoskeleton, the cell ensures that its structure can respond rapidly to changing cues. Such mechanical response may partly underlie the ability of the ER to restructure rapidly during mitosis, when it undergoes a global tubule-to-sheet conversion (72, 73).

Overall, the liquid network model not only accounts for the unique reticulated structure of the ER but also demonstrates how this architecture can emerge from and be regulated by a balance of two simple dynamical processes: tension-driven coarsening and new tubule growth.

## Materials and Methods

### Liquid Network Simulations.

Liquid networks are simulated via Brownian dynamics, integrating Eq. **1** forward in time. Length-minimizing T1 rearrangements are allowed to occur between colliding junctions, as described in *SI Appendix*.

### Cell Culture and Imaging.

COS7 and U2OS cells were cultured and transfected with mCh-KDEL or KDEL-venus (74). The peripheral ER of these cells was imaged using confocal microscopy (Zeiss LSM 880), as described in *SI Appendix*.

### Image Analysis.

The machine learning segmentation toolkit ilastik (75) is used to segment ER network structures and identify polygons in live-cell images. New tubule growth events are identified and tracked in a semiautomated protocol followed by manual verification. Details are described in *SI Appendix*.

### Quantifying Network Rearrangement.

Mean minimal distance between edges and polygon growth rates are computed to quantify network rearrangement dynamics, as described in *SI Appendix*.

## Supplementary Material

Appendix 01 (PDF)

Movie S1. Confocal movie of COS7 cell expressing fluorescent endoplasmic reticulum marker (KDEL_mcherry) demonstrating junction sliding and ring closure.

Movie S2. Four simulated liquid networks each with a different combination of junction mobility and tubule spawning rate leading to varying tubule density and rearrangement timescales. *b* = 10^−3^ μm/s and 10^−2^ μm/s in top and bottom row; *k* = 5 × 10^−4^ μm^−1^s^−1^ and 5 × 10^−3^ μm^−1^s^−1^ in left and right column, respectively.

Movie S3. Time evolution of polygon areas in a liquid network, highlighting growth and shrinking dynamics of the polygon population. Each circle represents the extracted area of a polygon centered at that location. Small circles shrink and large circles grow until they are split.

Movie S4. Liquid network simulation with semi-persistent pinning to static structures. Here, *b* = 0.02 μm/s, *k* = 0.005 μm^−1^s^−1^, *k_p_* = 10 s*−1*, *k_u_* = 0.01 s^−1^, leading to an average of 1000 pins at steady-state.

Movie S5. Example 6.3 × 6.3 μm region of peripheral ER, visualized for 40 s in order to count new tubule spawning events and calculate tubule spawning rate. Region is extracted from a confocal time-lapse image of COS7 cell expressing fluorescent endoplasmic reticulum marker (KDEL_mcherry). Video is shown in 2× real-time.

Movie S6. 3D liquid network simulation, with tubule contact distance *r* = 0.25 μm, *b* = 0.2 μm/s, *k* = 0.005 μm^−1^s^−1^, enclosed in a sphere of radius 10 μm. Movie is 500 s long.

## Data Availability

Software for liquid network simulations and a database of simulated and observed polygons are provided on GitHub (76).
