# Supplementary material for: The endoplasmic reticulum as an active liquid network
Source: Proc Natl Acad Sci U S A. 2024 Oct 11;121(42):e2409755121. doi: 10.1073/pnas.2409755121 (PMC11494354; doi:10.1073/pnas.2409755121)
Supplement: Supplementary file 1 — Appendix 01 (PDF) [file pnas.2409755121.sapp.pdf]

SUPPLEMENTARY MATERIAL: The Endoplasmic Reticulum as an Active Liquid Network

Zubenelgenubi C. Scott, Samuel B. Steen, Greg Huber, Laura M. Westrate, and Elena F. Koslover*

S1. LIQUID NETWORKS IN 3D

Here we briefly explore the extension of our liquid network model to three-dimensional (3D) systems. It should be noted that the peripheral ER in adherent COS7 cells is effectively a 2D network [1]; however, near the nucleus in adherent cells [1–3], and in the cell body of neuronal cells [4, 5], it can form a 3D networked structure.

A liquid network embedded in a 3D space has fundamentally different properties from the planar case. Specifically, it becomes necessary to consider a distinct length scale (r)—the effective contact distance within which a growing tip can fuse into the existing network structure. This distance is expected to be at least 0.1 µm, given the approximate 0.05 µm radius of COS7 ER tubules [6]. However, we may expect the contact distance to be substantially larger due to fluctuations, such as the thermal fluctuations of the tubules themselves (which have a typical persistence length of 8 µm [7]). Furthermore, ER tubules tend to be associated with the microtubule cytoskeleton [8–12], and growing tubules often hitchhike on other motor-driven organelles [13–16], which may affect the fluctuations of both the nascent and existing tubules. Here, we assume contact distances that range between $0.1 - 0.25$ µm, comparable to expected sizes of vesicular organelles swinging around microtubules.

We carry out simulations of 3D networks that are analogous to our 2D model (see Materials and Methods), with a few concrete changes. Specifically, we treat all tubules as spherocylinders, neglecting steric interactions, and allowing fusions to occur when the tip of a growing tubule comes within distance r of an existing edge. The direction of new tubule spawning is uniformly sampled from the set of directions perpendicular to the spawning edge.

All simulations take place in a sphere of radius $R_{\text{sim}} = 10$ µm. Growing nodes which reach R_{sim} cease their growth and become boundary nodes. These boundary nodes obey the same Langevin equation as other nodes, but their motion is restricted to sliding along the spherical boundary. If two boundary nodes come within the threshold distance (dx, just as in 2D) of one other and they share a neighbor, they annihilate and their common neighbor assumes their average position on the boundary. This allows for an analog of T1 rearrangements and loop closures at the boundary.

These simulations generate network structures (Fig. S1A, Video S6) that also reach a steady-state (Fig. S1B) whose density varies as a function of the balance between growth (k) and tension-driven relaxation (b). Given the introduction of a new length scale (r) for 3D networks, the scaling laws governing the steady-state geometry may be expected to differ from the 2D case. In particular, we look at the scaling of the average edge length λ versus the fundamental length scale $\ell = \sqrt{b/k}$. If we assume that the typical length that a new tubule must grow before fusing into the network scales in proportion to the average edge length λ, then in analogy to Eq. 2, we have the relationship:

$$\frac{dL}{dt} = \zeta k L \lambda - \gamma b n = 0 \tag{S1}$$

at steady-state. Neglecting numerical prefactors ζ, γ, and noting that the number of nodes scales as $n \sim L/\lambda$ gives the same predicted scaling relationship as in the 2D case: $\lambda \sim \ell$. Notably, if the average edge length is approximately independent of the total size of the domain, then dimensional analysis implies that it should not depend on any other length scale. Specifically, changing the contact distance r should have little effect on the average edge length. These predictions for the average edge length are borne out in our simulations (Fig. S1C).

* Author for correspondence: ekoslover@ucsd.edu (EFK)

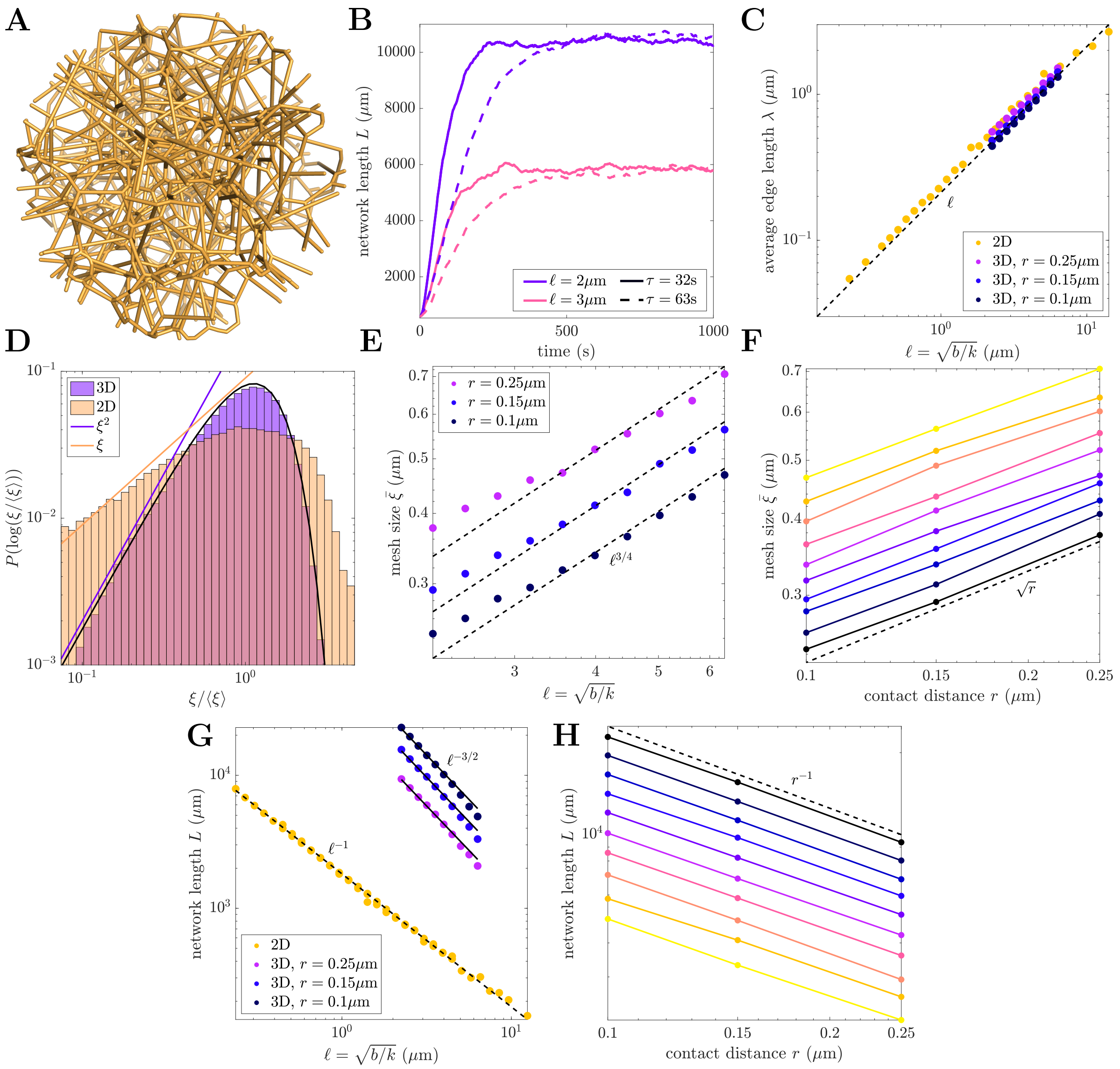

FIG. S1. Liquid network model produces stable structures in three dimensions, with steady-state geometries obeying different scaling laws than in two dimensions. (A) Example snapshot of a liquid network simulation within a sphere of radius 10 µm, with tubule contact distance $r = 0.25$ µm, and $\ell = 6.3$ µm. (B) Network length over time for four simulations with different fundamental length and time scales, $\ell = \sqrt{b/k}$ and $\tau = 1/\sqrt{bk}$, and $r = 0.25$ µm. The steady-state network length is dependent on ℓ while the time required to reach steady state is set by τ. (C) Average edge length scales linearly with ℓ for several tubule contact radii. Results from 2D networks shown in yellow for comparison; dashed black line shows $\lambda \sim \ell$ scaling. (D) Distributions of mesh sizes ξ in 2D and 3D liquid networks. Black line indicates fit to Rayleigh distribution with scale parameter $\sigma = 0.80$. Yellow and purple lines show approximate scaling for small mesh sizes. (E) Average mesh size $\bar{\xi}$ varies with length scale ℓ; dashed black lines indicate $\bar{\xi} \sim \ell^{3/4}$ scaling. (F) Mesh size scaling with contact distance; colors are different values of ℓ; dashed black line indicates $\bar{\xi} \sim r^{1/2}$. (G) Total network length L as a function of length scale $\ell = \sqrt{b/k}$ for several contact distances r. Dashed black line indicates ℓ^{-1} scaling for 2D networks (yellow); solid black lines indicate $\ell^{-1.5}$ scaling for 3D networks. (H) Total network length L scales inversely with tubule contact distance r, across a range of length scales ℓ (colors). Dashed black line indicates $L \sim r^{-1}$ scaling.

We next consider the mesh size of the network, analogous to the polygon size in the 2D case. Mesh size is determined by scattering random points ($N = 20000$) in the simulation volume and calculating the minimal distance from each point to the network [17]. The resulting distribution of mesh sizes is shown in Fig. S1D. For the 2D case, the small-size tail of the distribution scales according to $P(\log \xi) \sim \xi$, as expected from

Fig. 3A, given that $\xi \sim \sqrt{A}$. Interestingly, the 3D networks exhibit a distinctly different scaling for this small-size tail, with $P(\log \xi) \sim \xi^2$. The mesh size distribution for the 3D liquid networks is similar to the Rayleigh distribution observed in experimental studies on collagen and fibrin networks [17]. Notably, this distribution is also expected for 'Mikado' networks consisting of long, randomly distributed, disconnected lines of length much greater than the pore size [17].

The overall mesh size $\bar{\xi}$ for the network is defined as the average of this distribution. For a lattice-like network, the average mesh size is expected to scale with the average edge length, $\bar{\xi} \sim \lambda$. In an entangled, semi-flexible polymer network, there is an alternate scaling of $\bar{\xi} \sim \sqrt{\lambda r}$ [18]. In 3D liquid networks with a range of tubule contact distances, we find $\bar{\xi} \sim \ell^{3/4}$, falling between these two regimes (Fig. S1E). We also find $\bar{\xi} \sim \sqrt{r}$, matching the scaling expected for entangled, semi-flexible polymer networks (Fig. S1F) [18].

Putting these results together provides an estimate for how the total edge length L in the liquid network scales with the parameters. Namely, we would expect

$$L \sim \frac{R^3}{\bar{\xi}^2} \sim \ell^{-3/2} r^{-1}, \tag{S2}$$

which agrees with simulations in a fixed-size domain of radius $R = 10$ µm (Fig. S1G,H). In summary, increasing tubule radius has little effect on average edge length, but results in a spatially sparser network with larger pore size and less total edge length. Increasing $\ell = \sqrt{b/k}$ also leads to a sparser network, with a steeper scaling than in two dimensions.

More detailed exploration of 3D liquid network structure would require incorporation of explicit fluctuations in the ER tubules, which would be expected to increase the effective contact radius for fusion, possibly in a length-dependent manner. Furthermore, additional experimental characterization of 3D ER network structures is needed to establish typical pore sizes and edge lengths *in vivo*.

S2. STEADY STATE NETWORK PROPERTIES ARE INDEPENDENT OF JUNCTION DIFFUSIVITY AND MINIMAL LENGTH SCALE

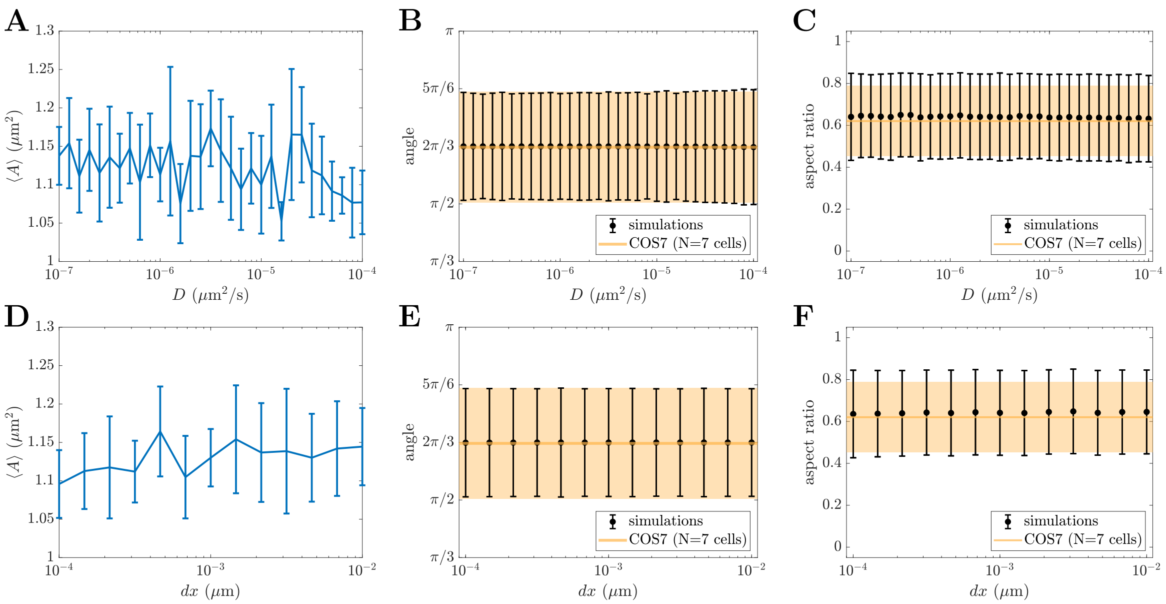

FIG. S2. Junction diffusivity and minimal length parameter do not affect liquid network properties. (A) Diffusivity of junctions is sufficiently small to have no effect on mean polygon area. Diffusivity has no effect on the mean and standard deviation of angles at three-way junctions (B) or the aspect ratio of polygons (C) Changing the distance within which junction rearrangements can occur (dx) does not substantially impact mean area of polygons (D), angles at three-way junctions (E), or the aspect ratio of polygons (F). All simulations performed with $b = 0.02$ µm/s, $k = 0.005$ µm^{-1}s^{-1}, and for (D)-(F), $D = 10^{-5}$µm^2/s.

In this section, we demonstrate that changes to junction diffusivity and the minimal length scale for junction rearrangements have no effect on steady state network properties, within a wide range around our chosen values.

Our choice for junction diffusivity (typically 10^{-5} μm^2/s) is sufficiently small to have no effect on polygon areas (Fig. S2A). Additionally, the mean and standard deviation of the distributions of angles at three way junctions and polygon aspect ratios are unaffected by junction diffusivity (Fig. S2B,C). The noise in these metrics instead arises from the random growth of new tubules and the tension-driven node sliding of polygons with a broad range of shapes.

In our simulations, $dx = \max(\sqrt{D \cdot dt}, b \cdot dt)$, sets the minimal length scale. When neighboring junctions are within this distance of one another, they check for possible rearrangements that would minimize the total connected edgelength. Steady state network properties, such as polygon area, junction angle and polygon aspect ratio, are insensitive to changes in dx when other simulation parameters are held fixed (Fig. S2D-F).

S3. POLYGON GROWTH DYNAMICS: REGULAR N-GONS

Informed by the equation of motion for junctions in a liquid network (Eq. 1), an approximate ansatz for the growth law can be derived. For simplicity, we consider the growth dynamics of regular n-gons, whose outgoing connections are assumed to bisect the angle of each junction. The rate of change in area A is given by:

$$\frac{dA}{dt} = \sum_{i=1}^{n} \frac{\partial A}{\partial \vec{r_i}} \cdot \frac{d\vec{r_i}}{dt} = n \frac{\partial A}{\partial \vec{r_1}} \cdot \frac{d\vec{r_1}}{dt} = n \left(\frac{\partial A}{\partial r_u} \right) \left(\frac{dr_u}{dt} \right). \tag{S3}$$

where $\vec{r_i}$ are the positions of each of the polygon's n identical junctions, and r_u is the component along the radial direction.

Assuming diffusion is negligible, the junction motion is determined solely by the length minimization term from Eq. 1, giving

$$\begin{aligned} \frac{dr_u}{dt} &= -b\frac{\partial f}{\partial r_u} = -b\left(2\sin\frac{\pi}{n} - 1\right) \\ \frac{dA}{dt} &= b\lambda n \left(\cos\frac{\pi}{n}\right)\left(1 - 2\sin\frac{\pi}{n}\right), \end{aligned} \tag{S4}$$

where λ is the side length of the polygon.

This predicts a negative growth rate for $3 \leq n < 6$ and a positive growth rate for $n > 6$. For small polygons, we would expect $n \approx 3$, and the shrinking rate should scale with the polygon perimeter ($\lambda n \sim \sqrt{A}$). For large polygons, the rate of growth can be described as $\frac{dA}{dt} \sim \sqrt{A} f(A)$ where $f(A)$ grows with the number of polygon sides. Because the number of sides is expected to grow with area, $f(A)$ should increase with A. We therefore make the approximation that large polygon growth rates increase as A^β for some approximate value of $0.5 < \beta < 1$. Overall, we take an ansatz that linearly combines the two scaling relations for small and large area polygons:

$$\frac{dA}{dt} = \hat{g}A^\beta - \hat{h}\sqrt{A}, \tag{S5}$$

where normalization by the average area yields Eq. 4 in the main text. It should be noted that this analysis does not account for the irregular shape of the polygons in the liquid network and thus provides only an approximation for the growth rate as a function of area.

S4. DERIVATION OF THE POLYGON AREA DISTRIBUTION FROM GROWTH AND SPLITTING LAWS

The goal here is to determine the steady-state distribution of polygon areas in a liquid network, $P(A)$, starting from the laws for growth and splitting of individual polygons and the assumption that polygons on

average take the form of a hexagon. The growth law extracted from simulations (Fig. 4) and the splitting rate of these polygons is expressed as:

$$\tau \frac{dA}{dt} = g\langle A\rangle^{1-\beta} A^{\beta} - h\sqrt{\langle A\rangle}\sqrt{A}, \tag{S6a}$$

$$k_{\text{split}} = \hat{k}\sqrt{A}, \tag{S6b}$$

where A is polygon area, $\langle A\rangle$ is the average area of all polygons, g, h, and β are constants determined from a fit of simulated growth rates, and $\hat{k} = (\sqrt{2\sqrt{3}})k$ is the prefactor for splitting in terms of the spawning rate k. Equation S6b is obtained by finding the perimeter p of a regular hexagon of area A and noting that the expected splitting rate is $k_{\text{split}} = \frac{1}{2}kp$, since new tubules can grow in either direction perpendicular to an edge.

If we make the additional assumption that polygons tend to split in half upon new tubule growth, the distribution of polygon areas, $P(A)$, would be expected to obey an effective deterministic Fokker-Planck equation:

$$\frac{\partial P}{\partial t} = -\frac{\partial}{\partial A}\left[\frac{dA}{dt}P(A)\right] - \hat{k}\sqrt{A}P(A) + 4\hat{k}\sqrt{2A}P(2A). \tag{S7}$$

Here, the first term corresponds to the area drift associated with polygon growth and shrinking, the second term describes polygon disappearance due to splitting, and the last term corresponds to the formation of new polygons after a splitting event [19]. We note that stochastic noise terms are neglected in this equation for simplicity. While such noise could be extracted from the breadth of the growth rate distribution in polygons of each size (Fig.4E), we choose to consider only the deterministic drift in polygon areas that shrink or grow in a size-dependent manner as the simplest possible description accounting for the observed steady-state area distribution.

S4A. Small area scaling

Although Eq. S7 is not easily solvable analytically, we can analyze its behavior in the limit of very small polygons. We consider a narrow band of small polygon areas: $(0, \delta A)$. The rate at which polygons disappear from this band due to shrinking below 0 area (J_0) must equal the total rate at which polygons enter the band. This includes the shrinking of larger polygons, which enter the band at rate $v(\delta A)P(\delta A)$ and the creation of new polygons due to splitting of polygons with area between $(0, 2\delta A)$:

$$J_0 = -v(\delta A)P(\delta A) + 2\hat{k}\int_0^{2\delta A}\sqrt{a}P(a)da - \hat{k}\int_0^{\delta A}\sqrt{a}P(a)da \tag{S8}$$

Here, the second term corresponds to 2 new polygons created by splitting and the last term accounts for the fact that the original polygon vanishes when a splitting event occurs. If we assume that the distribution function for small areas is a power law: $P(A) = A^{\mu}$, then the above equation can be expressed as:

$$J_0 = \hat{h}(\delta A)^{\mu+1/2} + \hat{k}\left(\frac{2^{\mu+5/2}-1}{\mu+\frac{3}{2}}\right)(\delta A)^{\mu+3/2} \tag{S9}$$

When very small areas are being considered ($\delta A \to 0$), the first term is dominant, so we have $J_0 \to \hat{h}(\delta A)^{\mu+1/2}$. In order to have a finite flux of disappearing polygons, countering the production of new polygons throughout the system, we must set $\mu = -1/2$.

Therefore the distribution function for polygons of small area can be written as

$$P(A) = \frac{c_1}{\sqrt{A}} \quad \text{(for small A)}, \tag{S10}$$

with c_1 a constant. Converting to a logarithmic distribution, $P(\log(A)) = AP(A) \sim \sqrt{A}$. This square-root scaling matches the distribution of small polygon areas measured for simulations of liquid networks (Fig. 3A and Fig. 4I).

S4B. Large area scaling

For very large values of the area A, the polygon dynamics are described by a balance between growth and splitting. We neglect the last term in Eq. S7, assuming that $P(2A) \ll P(A)$ so that the production of very large polygons by splitting events is rare. This assumption is self-consistent with an exponentially decaying distribution for large areas. Plugging the growth velocity from Eq. S6a into the truncated form of Eq. S7 gives the steady-state distribution of polygon areas as:

$$P(A) = c_3 c_1 A^{-\beta} e^{-c_2 A^z} \text{ for large A,} \tag{S11}$$

where $c_2 = \frac{\hat{k}}{gz} \langle A \rangle^{\beta - 1}$, $z = \frac{3}{2} - \beta$, and c_3, c_1 are constants.

S4C. Full distribution by stitching together the two limits

In order to construct the complete distribution, the two limits for small and large area are married together at some intermediate area A^*. Enforcing continuity of the distribution requires $c_3 = A^{*(1-z)} e^{c_2 A^{*z}}$ and gives the following functional form for the full distribution:

$$P(A) = \begin{cases} \frac{c_1}{\sqrt{A}} & \text{for } A < A^* \\ \frac{c_3 c_1}{A^\beta} e^{-c_2 A^z} & \text{for } A \geq A^* \end{cases} \tag{S12}$$

The remaining unknowns are c_1, A^*, and $\langle A \rangle$ (which also appears in c_2). In order to estimate A^* and $\langle A \rangle$, we first note that the total number of polygons must remain fixed at steady state. Thus, the flux of vanishing small polygons must equal the flux of newly created polygons via splitting: $J_0 = J_{\text{split}}$. The flux of vanishing polygons is:

$$J_0 \approx c_1 h \sqrt{\langle A \rangle} / \tau, \tag{S13}$$

and the flux of newly created polygons due to splitting is:

$$\begin{aligned} J_{\text{split}} &= \int_0^\infty \hat{k} \sqrt{A} P(A) dA \\ &= \int_0^{A^*} \hat{k} c_1 dA + \int_{A^*}^\infty \hat{k} c_3 c_1 A^{\frac{1}{2} - \beta} e^{-c_2 A^z} dA \\ &= \hat{k} c_1 A^* \left(1 + \frac{1}{c_2 z} (A^*)^{-z} \right) \end{aligned} \tag{S14}$$

Additionally, an expression for the average area is obtained via $\langle A \rangle = \int_0^\infty P(A) A dA / \int_0^\infty P(A) dA$. Through this matching of fluxes and the definition of average area, we obtain two equations:

$$\begin{aligned} h \sqrt{\langle A \rangle} / \tau &= \hat{k} A^* \left(1 + \frac{1}{c_2 z} (A^*)^{-z} \right), \\ \langle A \rangle &= \frac{\frac{2}{3} A^{*3/2} + \frac{c_3}{z c_2^{1 + 1/(2z)}} \Gamma \left(1 + \frac{1}{2z}, c_2 A^{*z} \right)}{2 \sqrt{A^*} + \frac{c_3}{z c_2^{1 - 1/(2z)}} \Gamma \left(1 - \frac{1}{2z}, c_2 A^{*z} \right)}, \end{aligned} \tag{S15}$$

where Γ is the incomplete Gamma function. These can be solved numerically for A^* and $\langle A \rangle$ using parameters g, h, and β from the effective growth law. Finally, the prefactor c_1 is set by normalizing the full distribution $\int_0^\infty P(A) dA = 1$.

Using the non-dimensionalized growth law extracted from liquid network simulations (Fig. 4E, Eq. 4), this gives $A^* \approx 0.12 \ell^2$ and $\langle A \rangle \approx 0.23 \ell^2$. We note that the predicted average area is similar to the actual value of $\langle A \rangle \approx 0.29 \ell^2$ obtained for simulated liquid networks (Fig. 2D).

The area A^* corresponds to the peak in the area distribution function $P(A)$ (see Fig. 4I) and thus gives an estimate of the most 'typical' polygon area. For COS7 ER, we have the length scale $\ell \approx 1.95$ µm, giving the predicted typical area $A^* \approx 0.46$ µm^2 in the liquid network model. For comparison, the peak in the measured area distribution for ER polygons in COS7 cells is 0.72 ± 0.32 µm^2 (mean and standard deviation across cells).

S5. POLYGON NEIGHBOR COUNTS, AREAS AND GROWTH RATES

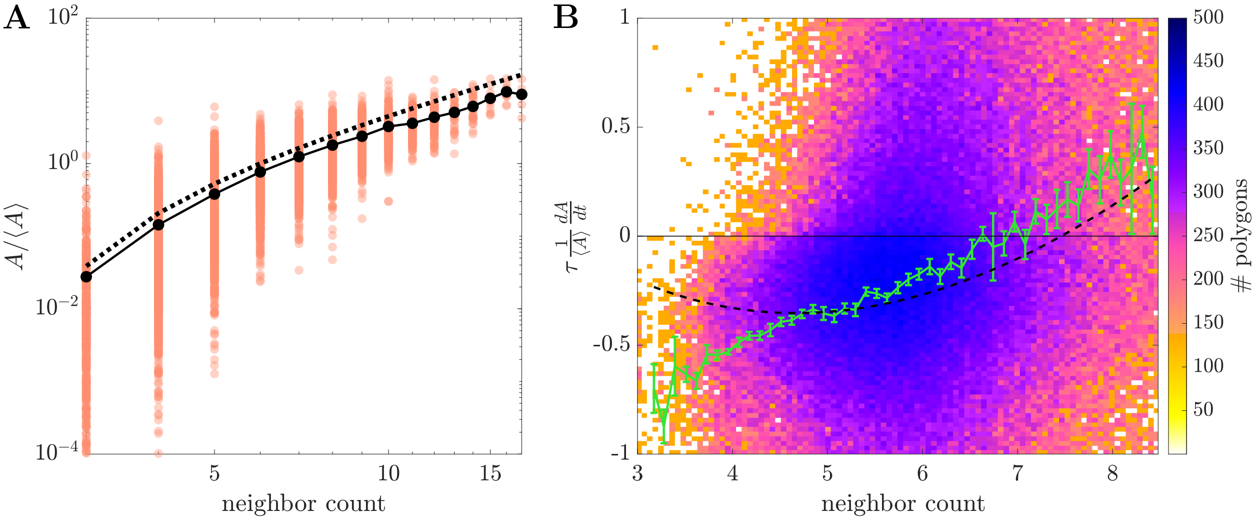

FIG. S3. Polygon area, neighbor count and growth rate are interconnected. (A) Polygon area as a function of neighbor count (same simulation data as in Fig. 4.) Solid black line gives mean area at each n, dotted black line is analytic prediction from Eq. S16. (B) Non-dimensionalized growth rates normalized by mean area and network timescale for 10 liquid network simulations. Green curve indicates mean (and standard error of the mean) within coarse bins of normalized neighbor count. Non-integer neighbor counts arise from averaging over 3 s intervals. Dashed black line is obtained by plugging Eq. S16 into Eq. 4, and using the fit parameters extracted from the growth rate vs area data (Fig. 4E).

In this section, we derive an approximate geometric relationship between a polygon's normalized area and its number of neighbors, and quantify the relationship between growth rates and neighbor count. To begin, we make the simplifying assumption of regular polygons, and compute the number of average-sized neighbor polygons that can be placed around a polygon of a given area. Specifically, we imagine a regular polygon with n sides, with outgoing connections bisecting its junction angles, surrounded by neighboring regular polygons with m sides. Note that m may not always be an integer, but it still provides an estimate for the expected relationship between side counts of the central and neighboring polygons. The internal angles of the neighbor polygons are given by $\theta = \pi\left(1 - \frac{2}{m}\right)$, which must be equated to half the external angle of the central polygon: $\theta = \frac{\pi}{n} + \frac{\pi}{2}$. This gives the relationship $m = \frac{4}{1-2/n}$.

The area of a regular polygon with side length ℓ can be expressed $A = n\ell^2/\left(4\tan\frac{\pi}{n}\right)$. Assuming that all neighbor polygons are average sized, with area $\langle A\rangle$, the normalized area of the central polygon can be expressed as:

$$\frac{A}{\langle A\rangle} = \frac{n\tan\frac{\pi}{m}}{m\tan\frac{\pi}{n}} = \left(\frac{n-2}{4}\right)\left(\frac{\tan\frac{\pi(1-2/n)}{4}}{\tan\frac{\pi}{n}}\right). \tag{S16}$$

This predicted scaling for area as a function of neighbor (or edge) count approximately captures the scaling of polygons in liquid networks (Fig. S3A). It should be noted that this derivation assumes regular polygons—for irregular polygons the area is expected to be slightly lower, as seen in the simulation data.

Polygon growth rates and the number of neighbors are intimately related. As in foams [20], regular polygons with more sides have a higher internal angle, translating to a net outward motion of junctions. We note that for liquid networks there is a large variation in the growth rate among polygons with the same number of neighbors (Fig. S3B). On average, however, the growth rate tends to scale together with the number of neighbors, with polygons that have 7 or more sides tending to grow, while those with fewer sides tending to shrink. The discrepancy from the classic von Neumann law (which predicts a cross-over from growth to shrinking at precisely 6 sides) may be due to the more irregular shape of liquid network polygons, and their tendency to change neighbor counts continuously as they grow.

Using the liquid network simulations, we can also demonstrate the consistency of the above formula for area as a function of neighbor count and the fitted ansatz for polymer growth rates vs area. Plugging Eq. S16 into Eq. 4, provides a predicted polygon growth rate as a function of edge count. Using the fit parameters from the main text for g, h, β and the values from fitting normalized area as a function neighbor count to Eq. S16 gives the dashed line in Fig. S3B. The two quantifications (growth as a function of area or growth as a function of neighbor count) appear to be relatively consistent for polygons with many neighbors. We note that the underestimate when converting to growth rate versus neighbor count may be the result of plugging averages from a noisy distribution into a nonlinear function. Polygons with 4 or fewer neighbors have limited data and thus may not provide an accurate estimate of the shrinkage rate. In the main text, we consider the dependence of polygon dynamics on the normalized area, both because of the tighter error bars and to allow for a direct derivation of the area distribution.

S6. BOUNDARY-FREE LIQUID NETWORKS

Here, we demonstrate that the boundary conditions employed in the main text have no effect on the steady-state properties of liquid networks. In particular, we show that liquid networks exhibit a well-defined steady-state density even in the absence of tethering to any static structures, including the outer boundary membrane.

To this end, we perform simulations of boundary-free liquid networks with a catastrophe rate of $\alpha = 0.25\ \text{s}^{-1}$, which allows for finite growth lengths of outwardly growing tubules. These networks expand their spatial footprint over time, forming complex webs evocative of *in vitro* images of reconstituted ER in Xenopus extract, which also show decreasing densities and longer tubules towards the network periphery [21].

There is no global steady-state in this system; the outer boundary continues to expand indefinitely. However, the architecture in the bulk of the network does reach a steady state. Network properties, such as average polygon area, within a region at the center of the network stabilize over time, and scale as expected with the kinetic parameters (Fig. S4C, Eq. 11).

This variant of the liquid network model demonstrates that the network is able to support tension and achieve a steady-state architecture without any connection to stationary structures. The length-minimizing tension is counteracted by the finite mobility of network nodes and the network density is ultimately determined by the balance between this mobility (b) and the rate of new tubule spawning (k).

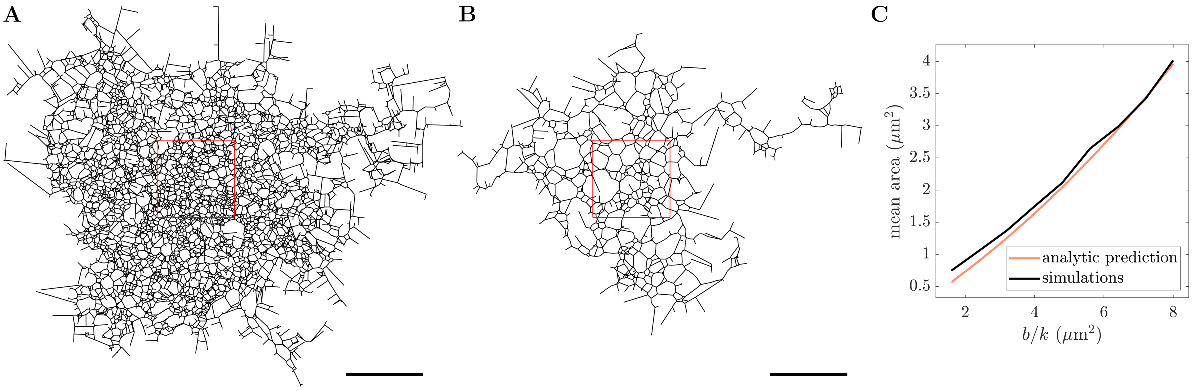

FIG. S4. Boundary-free liquid networks achieve steady state in the bulk. Example simulations after 1000s are shown with (A) low mobility, $b = 0.008$ µm/s and (B) high mobility, $b = 0.04$ µm/s. (C) Average polygon area calculated within central 20 µm × 20 µm region of boundary-free liquid networks, averaged across 10 simulations for each value of b/k. The orange curve indicates analytic prediction from Eq. 11 in the main text. In (A) and (B), scale bars and side lengths of red squares are 20 µm. Simulations performed with spawning rate $k = 0.005\ \mu\text{m}^{-1}\text{s}^{-1}$, catastrophe rate $\alpha = 0.25\ \text{s}^{-1}$, and over a range of mobilities $b = 0.004 - 0.04$ µm/s.

S7. MATERIALS AND METHODS

Liquid network simulations

Simulation code and a dataset of simulated polygon properties are provided at: `https://github.com/lenafabr/liquidNetworks`.

To simulate liquid networks, the overdamped Langevin equation for network nodes (Eq. 1) is integrated forward in time using a fourth-order Runge-Kutta method implemented in Fortran 90.

After updating junction positions in each time step dt, the code checks for connected nodes which are within some threshold distance of each other, defined to be $dx = \max(\sqrt{D \cdot dt}, b \cdot dt)$, where D is the diffusivity and b the mobility of the nodes. The threshold distance is thus comparable to the typical distance a node moves per time step, with values typically in the range of $dx = 10^{-4} - 10^{-3}$ µm. The model is insensitive to the specific choice of threshold (Fig. S2D-F).

For connected nodes within the threshold distance, the length of all possible rearrangements of the neighbors is calculated, to determine which configuration minimizes the connected length (Fig. 1D). The minimal configuration is chosen, and edges accordingly undergo their small rearrangements. Rearrangements are taken to occur within a single time step, consistent with the observation that rearrangement at ER junctions occurs at sub-second timescales, much faster than the timescales associated with polygon deformation.

Next, the code checks for fusion of actively growing tubule tips with existing edges by finding any crossings that may have occurred. Upon identification of a crossing event, the tubules fuse and form a stable junction node (Fig. 1D). Finally, the code samples from a Poisson distribution with rate k to determine the number of new tubule spawning events occurring in the current time step dt, given the current network length (excluding boundary edges). Novel spawning events occur uniformly distributed across all non-boundary edges of the network. Thus concludes one cycle of the simulation, after which the code iterates to update junction positions. Unless stated otherwise, a time step of $dt = 0.01$ s is used, the initial configuration is a honeycomb network, and the simulation is run until steady state has been reached (as defined by the stabilization of total network length over time).

To avoid runaway growth or full collapse of the membrane, all simulations are enclosed within a circle of radius 14 µm defined by fixed immobile junctions connected via boundary edges. When a tubule growth event fuses with a boundary edge, the resulting boundary junction behaves exactly the same as junctions in the bulk except its motion is restricted to the perimeter of the boundary circle. Neighbor rearrangements and loop closure can still occur, resulting in a net balance of boundary junctions over time. The circular boundary is taken to represent the plasma membrane, which is known to make direct contacts with the peripheral ER network.

Extensions to the model include random static pinning and unpinning of bulk nodes, and the catastrophe process for tubule growth, all modeled as random Poisson processes. Pinning of nodes occurs with rate k_p (s^{-1}), and unpinning of nodes occurs with rate k_u (s^{-1}). This pinning and unpinning is performed at the end of each simulation cycle. Catastrophe of each growing tubule occurs with rate α (s^{-1}). Catastrophe events are sampled every cycle after checking for tubule fusion. Boundary-free simulations are also performed, typically with a finite catastrophe rate for growing tubules. See Sec. S1 for model details in three dimensions.

We obtain spatial network structures over time from these simulations in the form of node positions and edge connectivity. To analyze the properties of minimal loops or polygons in the two-dimensional network structures, we use publicly available code written in Matlab [22].

Cell culture and imaging

COS7 cells (ATCC-CRL-1651) and U2OS cells (ATCC-HTB-96) were grown in Dulbecco's modified Eagle's medium (DMEM) supplemented with 10% fetal bovine serum (FBS) and 1% penicillin/streptomycin. Prior to imaging experiments, COS7 cells were seeded in six-well, plastic bottom dishes at a density of 150,000 cells per well, about 16 hours prior to transfection of DNA plasmids. The mCh-KDEL and KDEL-venus were described previously [23] and were transfected at a standard amount of 0.2 µg for all experiments. Plasmid transfections were performed 24 hours prior to imaging with plasmid DNA in Opti-MEM (Invitrogen) with 5 µL of Lipofectamine 3000 reagent (Invitrogen) according to the manufacturer's instructions. After 5 hours of transfection, cells were seeded in 35 mm glass-bottom microscope dishes (MatTek).

All imaging experiments were performed at the Van Andel Institute Optical Microscopy Core on a Zeiss LSM 880, equipped with an Axio Observer 7 inverted microscope body, stage surround incubation, Airyscan detector, two liquid-cooled MA_PMT confocal detectors, and one 32-channel GaAsP array confocal detector. Images were acquired with a Plan-Apochromat 63x (NA 1.4) oil objective with 3x optical zoom using Zeiss Zen 2.3 Black Edition software. Cells were tracked for at least 2 minutes with constant acquisition (200 ms or 316 ms).

Image analysis

A database of extracted polygon properties for COS7 cell peripheral ER is provided at: `https://github.com/lenafabr/liquidNetworks`.

Extracting networks from live cell images

The machine learning segmentation toolkit ilastik [24] is used to segment ER network structures from live-cell images using the KDEL_Venus or mCherry_KDEL markers. A network structure is extracted from the probability file output by ilastik using custom code for skeleton tracing in Matlab [25]. This code is publicly available at `https://github.com/lenafabr/networktools` and includes data structures for storing the network as a set of nodes connected by edges with curved spatial paths.

Estimating the tubule spawning rate in living cells

All new tubule spawning events are manually counted within cropped subregions (6.3×6.3 µm) of the peripheral ER from 19 cells over the course of 40 s (Video S5). The number of tubule spawning events is then normalized by the average network length over all frames (as obtained via ilastik and Matlab skeletonization, see above) and the total time considered. This yields an estimate for the average tubule spawning rate, k, (units of $\mu m^{-1} s^{-1}$) in each cell.

Extracting polygons from experimental ER images

The machine learning segmentation toolkit ilastik [24] is used to accurately identify polygons in experimental images of ER networks (7 COS7 cells and 2 U2OS cells). First, images are segmented as before using the pixel classification capabilities of ilastik. Then, an object classification model is trained to identify polygons in the cell periphery using the previously generated probability file and raw image as input. Associated geometric and spatial properties of these polygons are then analyzed in Matlab.

Semi-automated tracking of new tubule growth events

Network structures are extracted from images of COS7 cells, and all degree-1 (terminal) nodes are identified. The positions of these nodes and the length of their connected edges are calculated. The terminal nodes are then tracked over time using conventional particle tracking software [26] where the particle 'amplitude' is set by the connected edge length. Tracks within 1 µm of the network boundary or with track length less than 3 frames are discarded. The tracks are manually verified and adjusted as necessary using a custom-built Matlab GUI that superimposes track data on top of image files. The fate of the growing tubule (whether or not it successfully fuses into the network) is manually identified. A total of 200 tubule tip trajectories are quantified for Fig. 6.

The average velocity is found by dividing the maximum distance a tubule travels from its spawning point by the time it takes to get there. For successfully fused tubules, this is simply the distance from spawning to fusion; for unsuccessful spawning events, it is the distance from spawning to the turnaround or catastrophe point. The area and perimeter of the polygon containing the spawning event are calculated for each tracked frame. The gap size is estimated as $d = 4A/P$ and is averaged over the whole track.

Quantifying network rearrangement

Edge mean minimal distance

Both simulated and experimental networks are meshed into 0.03 µm segments, and for each point on subsequent networks, the minimal distance to a point on the starting network is found. The edge mean minimal distance (EMMD) is the mean of all such distances. To extract a timescale from the experimental data, the EMMD versus time curve is fit with a double exponential function of the form $f(x) = c_1(1-e^{-t/c_2})+c_3(1-e^{-t/c_4})$. The longer timescale (e.g. $c_2 \gg c_4$) is chosen as the representative EMMD timescale for each experimental network. Simulated data does not exhibit the same short timescale jump as experimental because there are no localization errors or network segmentation artifacts. Thus, only a single exponential (e.g. the first term of $f(x)$) is fit to the simulated EMMD curves, giving the extracted EMMD timescale for each simulated network.

Polygon growth rates

Experimental polygon positions and areas are extracted from ilastik segmented images (see above). Matlab File Exchange code for extracting minimal loops from graphs is used to find polygons in simulated networks [22]. Polygons are tracked over time using conventional particle tracking software [26]. For each tracked polygon, we also perform a change-point analysis of the tracked area over time using Matlab's findchangepts function [25]. This enables identification of additional splitting events that may have been missed by the tracking software. The tracks are broken up at the change-points, allowing for robust tracking of polygon growth and shrinking dynamics between split events.

From these tracks, the area rate of change over 3 s intervals is calculated in both experiments and simulations after applying a lowess smoothing filter with span 3 s. Only tracks longer than 4s are analyzed. The normalized growth rate is obtained by dividing the extracted growth rate by the average area of all tracked polygons in that cell or simulation run. In order to collapse together simulations with different parameter values, we multiply the simulated growth rate data by the intrinsic network timescale $\tau = (bk)^{-1/2}$, yielding a non-dimensionalized growth rate.

The mean and standard error of the mean for normalized growth rate are calculated within bins of normalized polygon area (green points in Fig. 4E, F). This provides an estimate for the average growth and shrinking behavior for polygons as a function of their area within liquid networks and the ER. The binned mean growth rates are then fitted to Eq. 4. For simulation data, β, g, h are the fitting parameters, whereas for experimental data we fix $\beta = 0.78$ (the scaling exponent obtained from the simulation fits) and only fit g, h. In both cases, the data is resampled 2000 times to estimate the error in the fitting parameters, assuming normally distributed values with mean and standard deviation obtained from binned values.

SUPPLEMENTAL VIDEO CAPTIONS

- **Video 1:** Confocal movie of COS7 cell expressing fluorescent endoplasmic reticulum marker (KDEL_mcherry) demonstrating junction sliding and ring closure.
- **Video 2:** Four simulated liquid networks each with a different combination of junction mobility and tubule spawning rate leading to varying tubule density and rearrangement timescales. $b = 10^{-3}$ µm/s and 10^{-2} µm/s in top and bottom row; $k = 5 \times 10^{-4}$ µm^{-1}s^{-1} and 5×10^{-3} µm^{-1}s^{-1} in left and right column, respectively.
- **Video 3:** Time evolution of polygon areas in a liquid network, highlighting growth and shrinking dynamics of the polygon population. Each circle represents the extracted area of a polygon centered at that location. Small circles shrink and large circles grow until they are split.
- **Video 4:** Liquid network simulation with semi-persistent pinning to static structures. Here, $b =$ 0.02 µm/s, $k =$ 0.005 µm^{-1}s^{-1}, $k_p =$ 10 s^{-1}, $k_u =$ 0.01 s^{-1}, leading to an average of 1000 pins at steady-state.
- **Video 5:** Example 6.3×6.3 µm region of peripheral ER, visualized for 40 s in order to count new tubule spawning events and calculate tubule spawning rate. Region is extracted from a confocal time-lapse image of COS7 cell expressing fluorescent endoplasmic reticulum marker (KDEL_mcherry). Video is shown in 2× real-time.
- **Video 6:** 3D liquid network simulation, with tubule contact distance $r =$ 0.25 µm, $b =$ 0.2 µm/s, $k =$ 0.005 µm^{-1}s^{-1}, enclosed in a sphere of radius 10 µm. Movie is 500 s long.

[1] J. Nixon-Abell, C. J. Obara, A. V. Weigel, D. Li, W. R. Legant, C. S. Xu, H. A. Pasolli, K. Harvey, H. F. Hess, E. Betzig, C. Blackstone, and J. Lippincott-Schwarz, Increased spatiotemporal resolution reveals highly dynamic dense tubular matrices in the peripheral er, Science **354**, aaf3928 (2016).

[2] L. Heinrich, D. Bennett, D. Ackerman, W. Park, J. Bogovic, N. Eckstein, A. Petruncio, J. Clements, S. Pang, C. S. Xu, J. Funke, W. Korff, H. F. Hess, J. Lippincott-Schwartz, S. Saalfeld, A. V. Weigel, and C. P. Team, Whole-cell organelle segmentation in volume electron microscopy, Nature **599**, 141 (2021).

[3] M. Puhka, H. Vihinen, M. Joensuu, and E. Jokitalo, Endoplasmic reticulum remains continuous and undergoes sheet-to-tubule transformation during cell division in mammalian cells, The Journal of cell biology **179**, 895 (2007).

[4] Y. Wu, C. Whiteus, C. S. Xu, K. J. Hayworth, R. J. Weinberg, H. F. Hess, and P. De Camilli, Contacts between the endoplasmic reticulum and other membranes in neurons, Proceedings of the National Academy of Sciences **114**, E4859 (2017).

[5] M. Elgendy, H. Tamada, T. Taira, Y. Iio, A. Kawamura, A. Kunogi, Y. Mizutani, and H. Kiyama, Dynamic changes in endoplasmic reticulum morphology and its contact with the plasma membrane in motor neurons in response to nerve injury, Cell and Tissue Research **396**, 71 (2024).

[6] T. Konno, P. Parutto, C. C. Crapart, V. Davì, D. M. Bailey, M. A. Awadelkareem, C. Hockings, A. I. Brown, K. M. Xiang, A. Agrawal, J. E. Chambers, M. J. Vander Werp, K. M. Koning, L. M. Elfari, S. Steen, E. Metzakopian, L. M. Westrate, E. F. Koslover, and E. Avezov, Endoplasmic reticulum morphology regulation by rtn4 modulates neuronal regeneration by curbing luminal transport, Cell Reports **43** (2024).

[7] H. T. Perkins, V. J. Allan, and T. A. Waigh, Network organisation and the dynamics of tubules in the endoplasmic reticulum, Sci Rep **11**, 16230 (2021).

[8] M. S. Tikhomirova, A. Kadosh, A. J. Saukko-Paavola, T. Shemesh, and R. W. Klemm, A role for endoplasmic reticulum dynamics in the cellular distribution of microtubules, P Natl Acad Sci **119**, e2104309119 (2022).

[9] J. Hu, W. A. Prinz, and T. A. Rapoport, Weaving the web of er tubules, Cell **147**, 1226 (2011).

[10] M. J. Wozniak, B. Bola, K. Brownhill, Y.-C. Yang, V. Levakova, and V. J. Allan, Role of kinesin-1 and cytoplasmic dynein in endoplasmic reticulum movement in vero cells, J Cell Sci **122**, 1979 (2009).

[11] J. R. Friedman, B. M. Webster, D. N. Mastronarde, K. J. Verhey, and G. K. Voeltz, Er sliding dynamics and er–mitochondrial contacts occur on acetylated microtubules, J Cell Biol **190**, 363 (2010).

[12] C. M. Waterman-Storer and E. Salmon, Endoplasmic reticulum membrane tubules are distributed by microtubules in living cells using three distinct mechanisms, Curr Biol **8**, 798 (1998).

[13] Y. Guo, D. Li, S. Zhang, Y. Yang, J.-J. Liu, X. Wang, C. Liu, D. E. Milkie, R. P. Moore, U. S. Tulu, D. P. Kiehart, J. Hu, J. Lippincott-Schwartz, E. Betzig, and D. Li, Visualizing intracellular organelle and cytoskeletal interactions at nanoscale resolution on millisecond timescales, Cell **175**, 1430 (2018).
[14] J. R. Friedman, J. R. DiBenedetto, M. West, A. A. Rowland, and G. K. Voeltz, Endoplasmic reticulum–endosome contact increases as endosomes traffic and mature, Mol Biol Cell **24**, 1030 (2013).
[15] M. Lu, F. W. van Tartwijk, J. Q. Lin, W. Nijenhuis, P. Parutto, M. Fantham, C. N. Christensen, E. Avezov, C. E. Holt, A. Tunnacliffe, D. Holcman, L. Kapitein, G. S. Kaminski Schierle, and C. F. Kaminski, The structure and global distribution of the endoplasmic reticulum network are actively regulated by lysosomes, Sci Adv **6**, eabc7209 (2020).
[16] J. R. Friedman, L. L. Lackner, M. West, J. R. DiBenedetto, J. Nunnari, and G. K. Voeltz, Er tubules mark sites of mitochondrial division, Science **334**, 358 (2011).
[17] N. R. Lang, S. Münster, C. Metzner, P. Krauss, S. Schürmann, J. Lange, K. E. Aifantis, O. Friedrich, and B. Fabry, Estimating the 3d pore size distribution of biopolymer networks from directionally biased data, Biophysical journal **105**, 1967 (2013).
[18] C. P. Broedersz and F. C. MacKintosh, Modeling semiflexible polymer networks, Reviews of Modern Physics **86**, 995 (2014).
[19] E. Khain and L. S. Tsimring, Effective pressure and cell area distribution in a confined monolayer, Fluid Dynamics Research **50**, 051413 (2018).
[20] J. von Neumann, Discussion: Shape of metal grains, Metal Interfaces **108** (1952).
[21] S. Wang, F. B. Romano, C. M. Field, T. J. Mitchison, and T. A. Rapoport, Multiple mechanisms determine er network morphology during the cell cycle in xenopus egg extracts, J Cell Biol **203**, 801 (2013).
[22] M. J, spatialgraph2d, `https://www.mathworks.com/matlabcentral/fileexchange/73630-spatialgraph2d` (2022), retrieved: May 1, 2022.
[23] A. R. English and G. K. Voeltz, Rab10 gtpase regulates er dynamics and morphology, Nat Cell Biol **15**, 169 (2013).
[24] S. Berg, D. Kutra, T. Kroeger, C. N. Straehle, B. X. Kausler, C. Haubold, M. Schiegg, J. Ales, T. Beier, M. Rudy, K. Eren, J. I. Cervantes, B. Xu, F. Beuttenmueller, A. Wolny, C. Zhang, U. Koethe, F. A. Hamprecht, and A. Kreshuk, ilastik: interactive machine learning for (bio)image analysis, Nat Methods 10.1038/s41592-019-0582-9 (2019).
[25] MATLAB, *version 9.11 (R2021b)* (The MathWorks Inc., Natick, Massachusetts, 2021).
[26] K. Jaqaman, D. Loerke, M. Mettlen, H. Kuwata, S. Grinstein, S. L. Schmid, and G. Danuser, Robust single-particle tracking in live-cell time-lapse sequences, Nat Methods **5**, 695 (2008).
